# An integrated SOX4-driven transcriptional and metabolic program governs tumor-infiltrating regulatory T cells in ovarian cancer

**DOI:** 10.7150/thno.131793

**Published:** 2026-08-12

**Authors:** Lei Wu, Ziqi Tao, Yuelu Zhang, Yepeng Mao, Lei Zhang, Lina Yan, Rong Li, Lingfei Zhou, Zhijie Liu, Wenwen Shang, Shuna Liu, Jianfang Lou, Xi Huang, Ting Wang, Fang Wang

**Affiliations:** 1 Department of Laboratory Medicine, The First Affiliated Hospital of Nanjing Medical University, No. 300 of Guangzhou Road, Nanjing, 210029, China.; 2 Branch of National Clinical Research Center for Laboratory Medicine, Nanjing, 210029, China.; 3 Department of Gynecology, The Affiliated Huaian NO.1 People's Hospital of Nanjing Medical University, Huaian, 223300, China.; 4 Department of Gynecology, Women's Hospital of Nanjing Medical University, Nanjing, 210004, China.

**Keywords:** regulatory T cells, ovarian cancer, tumor microenvironment, SOX4 transcription factor

## Abstract

**Background:**

Regulatory T cells (Tregs) suppress antitumor immunity in ovarian cancer (OC) and are promising targets for immunotherapy. However, the heterogeneity and regulatory mechanisms of tumor-infiltrating Tregs (TI-Tregs) remain poorly defined. Here, we aim to delineate TI-Treg programs to identify potential therapeutic targets.

**Methods:**

CD4⁺CD25⁺CD127⁻ Tregs from OC, adjacent tissues, and peripheral blood were profiled by single-cell RNA sequencing and spatial transcriptomics, with regulatory networks inferred using SCENIC. Functional and mechanistic studies of SOX4 were performed using hypoxic/tumor-conditioned models, CRISPR–Cas9 perturbation, ectopic overexpression, Cut&Tag profiling, and oxidative phosphorylation (OXPHOS) inhibition.

**Results:**

We identified nine transcriptionally distinct Treg subsets, revealing a highly activated and immunosuppressive state among TI-Tregs. These TI-Tregs exhibited strong co-expression of *TNFRSF4*, *TNFRSF9*, *TNFRSF18*, and *CTLA4*, and their increased intratumoral abundance was independently associated with poorer overall survival. SCENIC analysis identified SOX4 as the top regulon defining TI-Treg identity, with spatial transcriptomics revealing SOX4⁺ Tregs forming an immunoregulatory barrier at tumor margins. This phenotypic identity was robustly induced by the hypoxic microenvironment and TCR stimulation in a well-established murine OC model. Mechanistically, SOX4 transactivated MT1X to promote mitochondrial fitness and OXPHOS. Consistently, Cut&Tag profiling showed reduced chromatin accessibility at OXPHOS-related loci following SOX4 depletion. Furthermore, CRISPR–Cas9-mediated disruption of SOX4 reduced FOXP3 expression and other suppressive markers, whereas SOX4 overexpression enhanced FOXP3 expression in an OXPHOS-dependent manner. Notably, pharmacological inhibition of OXPHOS abolished this effect.

**Conclusions:**

SOX4 is a central regulator of TI-Treg suppressive function and metabolic fitness, representing a promising therapeutic target for OC.

## Introduction

Ovarian cancer (OC) remains particularly lethal gynecological malignancy due to its insidious onset, rapid progression, and the lack of effective early screening techniques 1. The overall 5-year survival rate for OC is approximately 40%, decreasing to 25% for patients diagnosed at stage III or IV. Although the clinical approval of bevacizumab and poly(ADP-ribose) polymerase inhibitors (PARPis) as first-line maintenance therapies has substantially improved outcomes in advanced OC, the long-term prognosis remains poor, with nearly half of patients dying from the disease within five years of diagnosis 2-4. Immune-checkpoint blockade has revolutionized cancer treatment in several solid tumor types, yet has been disappointing in OC, calling for a comprehensive search of critical immune factors 5, 6.

The ovarian cancer tumor microenvironment (TME) is commonly in an immune-suppressed state, marked by the accumulation of regulatory T cells (Tregs) and suppression of cytotoxic T cell responses. Other aspects include the enzymatic activity of indoleamine 2,3-dioxygenase (IDO), the interaction between PD-1/PD-L1, the presence of myeloid-derived suppressor cells and inhibitory cytokines such as transforming growth factor-β (TGF-β) and interleukin-10 (IL-10) 7. Targeting Treg mediated immunotolerance has become a major therapeutic focus in OC immunotherapy. Current translational advances are foucsed on four principal strategies: direct depletion of lineage-committed Tregs, modulation of co-stimulatory and co-inhibitory surface receptors, blockade of trafficking pathways to limit Treg infiltration into the TME, and induction of Treg functional fragility to alleviate local immunosuppression. However, the efficacy of Treg-targeted therapies in clinical settings is limited, mainly due to their inability to selectively target tumor-infiltrating Tregs (TI-Tregs/TIL-Tregs) and the impaired suppressive or anti-tumor effects of peripheral Tregs 6, 8.

In this study, leveraging single-cell RNA sequencing, we constructed a high-resolution atlas of OC–associated Tregs. We define the phenotypic features of TI-Tregs and, for the first time, identify SOX4 as a cardinal hallmark of this population, delineating its role and mechanism in reshaping TI-Tregs activation and inhibitory phenotypes. Zhang *et al*. 9 previously identified SOX4 in CD4^+^ T cells across various cancers, but their study was limited to bioinformatics analyses without any foundational experiments. Our analyses highlighted the regulatory roles of SOX4 with several surface markers differentially expressed on OC TI-Tregs, including *TNFRSF4* (OX40), *TNFRSF9* (4-1BB), *TNFRSF18* (GITR), *CTLA4* (CTLA-4), *ICOS* (CD278), PDCD1(PD-1) and LAG3(CD223). These findings unveil a unique transcriptional signature of TI-Tregs associated with poor prognosis, identify distinct surface markers that define functionally heterogeneous Tregs within the TME, and provide new insights into potential immunotherapeutic targets for OC.

## Methods

### Research design

This study aims to characterize the transcriptional landscapes of Treg populations across peripheral blood, peri-tumoral regions, and tumor tissues from both healthy individuals and patients with OC. We further seek to define the molecular features of tumor-infiltrating Treg subpopulations and their associations with clinical outcomes. In addition, we investigate the regulatory role of the transcription factor (TF) SOX4 and its downstream gene network in sustaining the suppressive phenotype of OC associated TI-Tregs. Blood, peri-tumoral, and tumor samples from OC patients were obtained after approval from the Ethics Review Committee of the First Affiliated Hospital of Nanjing Medical University. All patients provided written informed consent at both the First Affiliated Hospital of Nanjing Medical University and Nanjing Maternal and Child Health Hospital. Blood from healthy individuals was voluntarily provided, with participants providing written consent and receiving compensation. **Table S1** summarizes the clinical characteristics of all participating OC patients, including age, tumor size, histological type, stage, grade, and presence of lymph node and distant metastases. We performed scRNA-seq on Tregs infiltrating the tumor tissues of the three patients, the peri-tumoral tissue of one patient, and the peripheral blood of the OC patient, as detailed in **Table S2**, using Tregs sorted from healthy peripheral blood as controls to characterize the unique transcriptional features and cellular states of the patient-infiltrating Tregs. Key Treg subgroups correlated with patient survival outcomes were identified using BayesPrism and TCGA survival analysis. We also applied SCENIC to this key effector subgroup to identify highly active regulatory TFs, including SOX4, and to construct a downstream target gene network. Bioinformatics analysis of the GSE184880 scRNA-seq dataset validated the Treg gene set constructed from our study data as applicable to public data, accurately distinguishing OC TI-Tregs from other immune cell subgroups. Gene set enrichment analysis was used to identify pathways enriched in OC tissues and effector Treg subgroups. Flow cytometry was then used to validate the high expression of activation and suppression phenotype molecules in tumor-infiltrating Tregs in OC. RT-PCR and flow cytometry were also used to verify the expression of TFs such as SOX4 at the transcriptional and protein levels in peripheral blood and tumor tissue cells, including CD8^+^ T cells, Tconv, and Tregs. Small interfering RNA (siRNA) technology was employed to interfere with the SOX4 gene in human activated Tregs, and flow cytometry was used to measure the expression levels of FOXP3 and PD-1 in the control and gene knockdown groups.

### Peripheral blood and tissue sample processing

Peripheral blood mononuclear cells (PBMCs) were isolated using density gradient centrifugation with Ficoll. Tissue samples, including fresh ovarian tumor tissues or peri-tumoral tissues, were minced into 1 mm³ pieces and digested in a custom digestion solution (RPMI-1640 medium, collagenase IV (1 μg/mL), hyaluronidase (100 ng/mL), DNase I (50 U/mL), glutamine (1 mM) and 1% penicillin-streptomycin solution) at 37 °C on a shaker for one hour and then filtered through a 40 μm strainer. The filtered cell suspension was subjected to 70% and 30% Percoll gradient centrifugation to obtain single-cell suspension. Live CD4^+^CD25^+^CD127^-^ Tregs were sorted from these suspensions using flow cytometry.

### Single-cell RNA library preparation and sequencing

Single-cell suspensions (2×10^5 cells/mL) were mixed with PBS (HyClone) and loaded onto a microfluidic chip using the Singleron Matrix® single-cell system 10. Barcode beads were collected from the microchip for mRNA reverse transcription, captured by barcode beads to obtain cDNA, and amplified by PCR. The amplified cDNA was then fragmented and ligated to sequencing adapters. The scRNA-seq libraries were constructed using the GEXSCOPE® Single-cell RNA Library Construction Kit from Singleron according to the protocol. Each library was diluted to 4 nM, pooled, and sequenced on an Illumina NovaSeq 6000 with 150 bp paired-end reads 11.

### Normalization and integration of single-cell RNA sequencing data

Cells with fewer than 200 detected genes or mitochondrial counts > 40% or ribosomal counts > 50% were removed; cells with genes detected in three or more cells were retained; cells with gene counts > 1000 were kept. The Scrublet algorithm was run to eliminate any potential doublets, with an expected doublet rate set at 0.05. The original datasets were integrated using "Harmony" to mitigate batch effects and facilitate unified downstream analysis. Cells in the obtained two-dimensional representation were annotated with canonical marker genes to known biological cell types 12.

### Identification and functional analysis of differentially expressed genes

The "Find All Markers" function in Seurat 3.1 13, 14 was used to perform Wilcoxon rank-sum tests to identify differentially expressed genes in each cell cluster 15. Differentially expressed genes were defined as those expressed in a proportion of cells ≥ 0.25 in one group, with an average expression level at least 0.25 times higher in one cell subgroup compared to others.

### Deconvolution of cell composition using single-cell RNA Sequencing data

Data from GSE184880, which performed deep single-cell RNA sequencing on 59,324 cells isolated from seven untreated high-grade serous ovarian cancer (HGSOC) patients (at early or late stages) and five age-matched non-malignant ovarian samples, were downloaded from the Gene Expression Omnibus (GEO) official website 16. Bulk RNA-sequencing data and corresponding clinical annotations for 379 patients with ovarian serous cystadenocarcinoma from The Cancer Genome Atlas (TCGA-OV) were retrieved through the NCI Genomic Data Commons (GDC). Expression levels were normalized to Transcripts Per Kilobase of exon model per Million mapped reads (TPM). Random sampling of each subgroup in the integrated single-cell RNA sequencing data (500 cells per subgroup) was used to construct specific gene expression profiles for different cell types, then BayesPrism was applied to the normalized OC matrix for deconvolution to obtain the proportions of different cell types in each sample 17.

### Clinical survival prognosis analysis

Differences between the high-expression and low-expression groups post-BayesPrism deconvolu-tion were analyzed using the Wilcoxon rank-sum test or two-sided Student-t test. The Kruskal-Wallis test was used for analyzing differences between multiple paired comparisons. Overall survival rates were assessed through Kaplan-Meier curves and Cox regression analysis. A* P*-value < 0.05 was considered statistically significant.

### Pseudo-bulk differential expression analysis between different tissues

Transcript counts for each gene in each subgroup from four sample types (healthy peripheral blood, OC patient peripheral blood, peri-tumoral, and tumor tissues) were summed to generate pseudo-bulk samples 18, and differential analysis was conducted using DESeq2 (3.18) 19. Pseudo-bulk samples consisting of fewer than ten cells were removed, and multiple testing correction was used to control the false discovery rate.

### Spatial transcriptomics deconvolution

Public spatial resolved transcriptomic profiles from two OC samples (Visium_FFPE_Human_Ovarian_Cancer, CytAssist_11mm_FFPE_Human_Ovarian_Carcinoma) were retrieved and downloaded directly from the official 10x Genomics dataset repository. The cell2location model is a Bayesian framework that estimates the absolute abundance of cell types at each spatial location by decomposing the spatial gene expression count matrix into predefined reference signatures for each cell type 18. It utilizes an untransformed spatial gene expression count matrix d*_s,g_*, where *s* denotes spatial locations or spots and *g* denotes genes. For 10x Genomics Visium data, this matrix is typically generated from the Space Ranger output 19. The cell2location method also supports joint modeling of multiple spatial transcriptomic datasets for batch integration across independent experiments and different spatial transcriptomics technologies 20, 21. The model further requires a matrix of reference cell-type signatures g*_f,g_*, which reflects the expected mRNA counts of gene *g* for each cell type *f* 20, 21. This reference matrix is usually estimated using a negative binomial regression model, facilitating the integration of data across different batches and technologies. To further validate the inferred cell-type localization and spatial niches within the OC microenvironment, we used high-resolution Xenium *In Situ* data generated from the 10x Genomics platform (Xenium_Prime_Human_Ovary_Cancer_FF).

### Tregs isolation and culture

Single-cell suspensions obtained from peripheral blood and tissue samples were stained with antibodies (FVS780, Anti-CD4-FITC, Anti-CD25-APC, Anti-CD127-PE) and sorted for live CD4^+^CD25^+^CD127- Tregs using the BD FACS Aria II SORP high-speed flow cytometer. These flow-sorted Tregs were subsequently subjected to single-cell RNA sequencing and analysis. Bulk Treg cells for basic experiments were obtained using the CD4^+^CD25^+^CD127dim/- Regulatory T Cell Isolation Kit (Miltenyi Biotec, 130-094-775) with magnetic beads, and their purity was also verified through flow analysis.

### Tumor microenvironment model construction

CD4^+^CD25^+^CD127^-^Tregs isolated from healthy peripheral blood using magnetic beads were cultured in 96-well round-bottom plates at a density of 20,000 cells/well in 200 µL of X-VIVO15 medium, with an equal volume of CD3/CD28 expansion beads and 1000 U/mL IL-2, in a hypoxic environment (1.5% O_2_, 5% CO_2_, 92.5% N_2_) for 5 days 22.

### Detection of Treg cell surface markers

After isolating single-cell suspensions, 1×10^6^ single cells were added to flow tubes, washed twice with PBS, and centrifuged to discard the supernatant. 1 µL of flow antibody FVS780 was added to each tube, along with 5 µL of the respective flow antibodies Anti-CD4-FITC, Anti-CD25-BV421, Anti-CD127-PE, Anti-CD8-FITC, Anti-OX40-BV605, Anti-4-1BB-APC, Anti-GITR-BB700, Anti-PD-1-APC, Anti-TIM-3- PE/Cyanine7, and Anti-LAG-3-APC, shaken to mix, and incubated in the dark at 4 °C for 30 min (Appropriate flow cytometry antibodies listed in **Table S3**). After surface marking, tubes were washed twice with PBS and made into 200 µL cell suspensions per tube for analysis on the Beckman CytoFlex instrument.

### Detection of intracellular transcription factors in Tregs

1×10^6^ single cells were added to flow tubes, washed twice with PBS, and centrifuged at 1500 rpm for 5 min to discard the supernatant. 1 µL of flow antibody FVS780, 5 µL Anti-CD4-FITC, 5 µL Anti-CD25-BV421, and 5 µL Anti-CD127-PE were added to each tube, shaken to mix, and incubated in the dark at room temperature for 20 min, then washed with PBS. A lysis solution was prepared by mixing 4× Fix/Perm Buffer and Diluent Buffer at a 1:3 ratio, 1 mL added to each tube, shaken to mix, and incubated in the dark at 4 °C for 45-60 min; a washing solution was prepared by mixing 5× Perm/Wash Buffer and deionized water at a 1:4 ratio, washed twice, then centrifuged at 2300 rpm for 6 min and the supernatant discarded. 10 μL of flow antibody Anti-FOXP3-PE and 5μL Anti-SOX4-Alexa Fluor405 were added, shaken to mix, and incubated in the dark at 4 °C for 30 min; washed twice with washing solution, then made into 200 µL cell suspensions per tube for analysis on the Beckman CytoFlex instrument.

### Cas9 RNP assembly and transfection

EasyEdit sgRNAs targeting SOX4 were synthesized as follows: SOX4 sgRNA #1, 5′-GCTGGTGCAAGACCCCGAGT-3′; SOX4 sgRNA #2, 5′-AGGAGGCGATTCCCAGCTCG-3′; SOX4 sgRNA #3, 5′-AAAGAGGCGCGAGGCGGAAT-3′; SOX4 sgRNA #4, 5′-GCCGCGCGCGTCTTCCCGTT-3′; and scrambled control #1, 5′-CATATTGCGGGTATAGTCGC-3′. The EasyEdit sgRNAs were thoroughly mixed with Cas9 protein from the ProteanFect CRISPRMAX Ultra Transfection Kit (Nanoportal Biotech, PT08) at a ratio of 2:1 and incubated at 37°C for 15 min to form Cas9 ribonucleoprotein complexes (RNPs) 23. Human Tregs expanded under hypoxic conditions for 5 days were seeded into 24-well plates 24 h before transfection at a density of 1 × 10⁵ cells per well. On the day of transfection, Cas9 RNPs were delivered into Tregs using transfection reagents A–C from the ProteanFect CRISPRMAX Ultra Transfection Kit according to the manufacturer’s instructions, followed by incubation at 37 °C. Cells were harvested 24 h after transfection for transcript-level detection and analysis, whereas cells collected 48–72 h after transfection were used for protein-level detection and analysis.

### Mouse experiments

All animal experiments were conducted with the approval of the Animal Ethics Committee of Nanjing Medical University (Ethics Approval Number: IACUC-2303001). The ID8 cell line, a surface epithelial OC cell line from C57BL/6 mice, was purchased from Shenzhen Otwo Biotechnology Co., Ltd. Nine C57BL/6 mice were obtained from Nanjing GemPharmatech LLC. After thawing, 1×10^7^ ID8 cells were passaged three times and prepared as a 100 µL cell suspension. The suspension was mixed with 100 µL of a high-concentration matrix gel containing 20 mg/mL protein, and the mixture was injected subcutaneously into the lateral abdominal area of the mice. The mice were maintained under SPF conditions for 8 weeks post-injection before being euthanized by CO₂ asphyxiation. Spleens and tumor tissue samples were collected. The spleen tissues were ground to prepare single-cell suspensions, and red blood cell lysis buffer was used to isolate mononuclear cells from the spleen samples. Fresh subcutaneous ovarian tumor tissues were minced into 1mm³ pieces and digested in a primary tissue digestion solution (RPMI-1640 medium supplemented with collagenase IV (1 µg/mL), hyaluronidase (100 ng/mL), DNase I (50 U/mL), glutamine (1 mM), and 1% penicillin-streptomycin solution) at 37 °C in a shaker for 1 hour. The digested mixture was then filtered through a 40 µm filter, and the resulting cell suspension was subjected to gradient centrifugation with 80% and 20% Percoll to obtain a mononuclear cell suspension. Mononuclear cell suspensions from the spleen, peripheral blood, and tumor tissues of the mice were plated into flow cytometry tubes. Appropriate flow cytometry antibodies (listed in **Table S3**) were added. After incubation, lysis, washing, and fixation, the samples were analyzed using a Beckman CytoFLEX flow cytometer.

### Lentiviral transduction of SOX4 in human CD4^+^ T cells

Human PBMCs were isolated from peripheral blood, and CD4+ T cells were purified using magnetic bead-based separation. Purified CD4^+^ T cells were activated with anti-CD3 antibody at 5 μg/mL and anti-CD28 antibody at 3 μg/mL for 48 h. For lentiviral transduction, lentiviral particles and Polybrene were thawed on ice and mixed with X-VIVO 15 medium supplemented with 5% human AB serum and 500 IU/mL IL-2 to prepare the viral transduction mixture. The multiplicity of infection (MOI) was set at 100, and the required viral volume was calculated according to the following formula: viral volume (μL) = MOI × cell number × 10³ / viral titer (TU/mL).

A total of 1 × 10⁵ activated CD4^+^ T cells were resuspended in 200 μL of viral transduction mixture and seeded into each well of a 96-well flat-bottom plate. The plate was sealed and centrifuged at 1,000 g for 90 min with slow acceleration and deceleration. After centrifugation, cells were cultured at 37 °C in a humidified incubator containing 5% CO₂ for 24 h. The viral transduction mixture was then removed and replaced with fresh complete T-cell culture medium. Cells were cultured for an additional 48 h before collection for subsequent experiments.

### Quantitative real-time PCR analysis of MT1X mRNA expression

SOX4-overexpressing CD4^+^ T cells (SOX4-OE) and control vector-transduced CD4^+^ T cells (Vector) were collected 48 h after lentiviral transduction. Total RNA was extracted using TRIzol reagent (Invitrogen), and reverse transcription was performed using HiScript II Q Select RT SuperMix for qPCR (Vazyme, R232-01) according to the manufacturer’s instructions. Quantitative real-time PCR was performed on an ABI 7500 Real-Time PCR System (Applied Biosystems) in a 20 μL reaction system containing cDNA, gene-specific primers, and AceQ qPCR SYBR Green Master Mix (Vazyme, Q121-02). The expression level of each target gene was calculated using the standard curve method and normalized to HPRT expression.

### Measurement of MT1X by competitive ELISA

MT1X protein levels were measured using an in-house competitive enzyme-linked immunosorbent assay. Briefly, high-binding 96-well microplates were coated overnight at 4 °C with recombinant human MT1X protein (Proteintech, Ag10711) diluted in carbonate-bicarbonate coating buffer. After washing with PBST, plates were blocked with 1% BSA in PBS for 1 h at room temperature. Recombinant MT1X standards and diluted samples were pre-incubated with anti-MT1X rabbit polyclonal antibody (17172-1-AP, Proteintech) for 1 h at room temperature, and the mixtures were then added to the MT1X-coated plates. After incubation and extensive washing, HRP-conjugated goat anti-rabbit IgG was added and incubated for 1 h at room temperature. The reaction was developed with TMB substrate and stopped with sulfuric acid stop solution. Absorbance was measured at 450 nm with wavelength correction at 570 or 630 nm. MT1X concentrations were calculated from a four-parameter logistic standard curve and corrected for sample dilution. All samples and standards were assayed in duplicate or triplicate.

### Multiplex immunohistochemistry (mIHC) staining

Formalin-fixed, paraffin-embedded (FFPE) tissues obtained from 5 OC patients were sectioned at 2–4 μm thickness. After deparaffinization and heat-induced antigen retrieval using citrate or EDTA buffer, slides were blocked and sequentially incubated with primary antibodies targeting panCK, CD4, CD25, and SOX4, followed by tyramide signal amplification (TSA)-based fluorescent labeling according to the manufacturers’ instructions. Nuclei were counterstained with DAPI and mounted with antifade medium. Fluorescence images were acquired using Akoya Vectra, Nikon C1, or Leica TCS-SP8 systems. Spectral unmixing and quantitative image analyses were performed using Akoya inForm, LAS X, and ImageJ software.

### Mouse tumor model and treatment

On day 0, C57BL/6 mice were subcutaneously inoculated with murine ID8 OC cells in the unilateral axillary region at a dose of 1×10^7^ cells per mouse. Seven days after tumor inoculation, mice received the first intraperitoneal administration of anti-PD-1 antibody (150 μg per injection), rotenone (0.5 mg/kg), isotype control antibody (150 μg per injection), or vehicle control consisting of 10% DMSO and 90% corn oil (100 μL per injection). All treatments were administered intraperitoneally every 3 days on days 7, 10, 13, 16, 19, 22, 25, 28, 31, 34, 37, 40, 43, 46, 49, 52, and 55. Tumor volume was measured once every 7 days during the treatment period. On day 56, mice were euthanized, and tumor tissues, peripheral blood, lymph nodes, heart, liver, spleen, lung, kidney, and brain tissues were collected for subsequent analyses.

### Western blotting

SOX4-overexpressing (SOX4-OE) and vector-control CD4⁺ T cells were collected 72 h after lentiviral transduction. A total of 1 × 10⁶ cells from each group were washed with PBS and lysed in RIPA buffer supplemented with 1% protease inhibitor, 1% phosphatase inhibitor, and 0.5% PMSF. Total protein was extracted, and protein concentrations were determined using a micro-spectrophotometer. Equal amounts of protein were separated by 12% SDS-PAGE and transferred onto PVDF membranes. Membranes were blocked with 5% BSA at room temperature for 2 h and then incubated overnight at 4 °C with recombinant anti-Total OXPHOS antibody cocktail diluted at 1:1000. After washing with TBST, membranes were incubated with HRP-conjugated goat anti-rabbit IgG (H+L) secondary antibody diluted at 1:1000 for 1 h at room temperature. Protein bands were visualized using an enhanced chemilumi-nescence detection system. The membranes were then stripped and reprobed with anti-β-actin antibody overnight at 4 °C, followed by incubation with HRP-conjugated secondary antibody and chemilu-minescent detection.

### Rotenone treatment

SOX4-OE and vector-control CD4⁺ T cells were collected 72 h after lentiviral transduction. For each group, 5 × 10⁵ cells were washed with PBS, resuspended in 1 mL complete T-cell culture medium, and seeded into 24-well plates. Cells were treated with rotenone at a final concentration of 100 nM or with an equal volume of DMSO as vehicle control. Four experimental groups were established: SOX4-OE plus rotenone, SOX4-OE plus DMSO, vector plus rotenone, and vector plus DMSO. Cells were cultured at 37 °C in a humidified incubator containing 5% CO₂ for 72 h and then collected for subsequent analyses.

### Flow cytometric analysis of CD4⁺ T cells after rotenone treatment

After 72 h of rotenone or DMSO treatment, cells from the four experimental groups were collected for flow cytometric analysis. A total of 1 × 10⁶ cells per sample were washed with PBS and stained with fixable viability dye diluted at 1:1000 in PBS for 30 min at 4 °C. After washing, cells were stained with APC-conjugated anti-human CD25, PE/Cyanine7-conjugated anti-human CTLA-4, BV650-conjugated anti-human PD-1, and PerCP-Cy5.5-conjugated anti-human GITR antibodies for 30 min at 4 °C. Cells were then fixed and permeabilized using a Transcription Factor Buffer Set according to the manufacturer’s instructions, followed by intracellular staining with PE-conjugated anti-human FOXP3 antibody for 45 min at 4 °C. After washing, cells were resuspended in PBS and analyzed using a Beckman CytoFLEX flow cytometer.

### Flow cytometric analysis of surface markers, transcription factors, intracellular cytokines, and OXPHOS-related parameters in CD4⁺ T cells

CD4⁺ T cells were washed with PBS and stained with fixable viability dye and antibodies against surface markers, including CD25, PD-1, CTLA-4, GITR, and OX40, for 30 min at 4 °C in the dark. Surface staining was stopped by washing the cells with PBS. For TF analysis, intracellular FOXP3 staining was performed after fixation and nuclear permeabilization using a BD transcription factor staining buffer set according to the manufacturer’s instructions.For intracellular cytokine detection, cells were stimulated with 50 ng/mL PMA, 1 μg/mL ionomycin, and 10 μg/mL brefeldin A for 4 h before staining. After surface staining, cells were fixed and permeabilized, followed by intracellular cytokine staining according to the manufacturer’s protocol.For detection of mitochondrial respiratory chain complexes, cells were fixed and permeabilized using a BioLegend intracellular staining buffer set according to the manufacturer’s instructions. Cells were then incubated with recombinant anti-Total OXPHOS antibody cocktail diluted at 1:500 in PBS for 40 min at room temperature in the dark. After washing with PBS, cells were incubated with goat anti-rabbit IgG H&L secondary antibody diluted at 1:2000 in PBS for 40 min at 4 °C in the dark. The staining reaction was stopped by washing with PBS.For the detection of ATP, total ROS, mitochondrial ROS, and mitochondrial mass, cells were incubated with the corresponding fluorescent probes at 37 °C for 30 min in the dark. After staining, cells were washed with PBS and analyzed by flow cytometry.

### Statistical analysis

Statistical analyses were performed to assess differential gene expression, survival associations, and gene co-expression patterns. Differential expression analysis was conducted using the Wilcoxon rank-sum test. Survival outcomes were evaluated using Cox proportional hazards models in both univariate and multivariate settings. Pearson correlation analysis was applied to examine gene co-expression relationships and Treg signature-related profiling. These analyses were carried out in R software (version 4.3.1). For flow cytometry data, statistical significance was determined using two-way ANOVA, the non-parametric Mann–Whitney U test, and paired ratio t-tests, implemented in GraphPad Prism (version 9).

## Results

### Single-cell characterization of tumor-infiltrating Tregs in ovarian cancer

**Figure 1:**
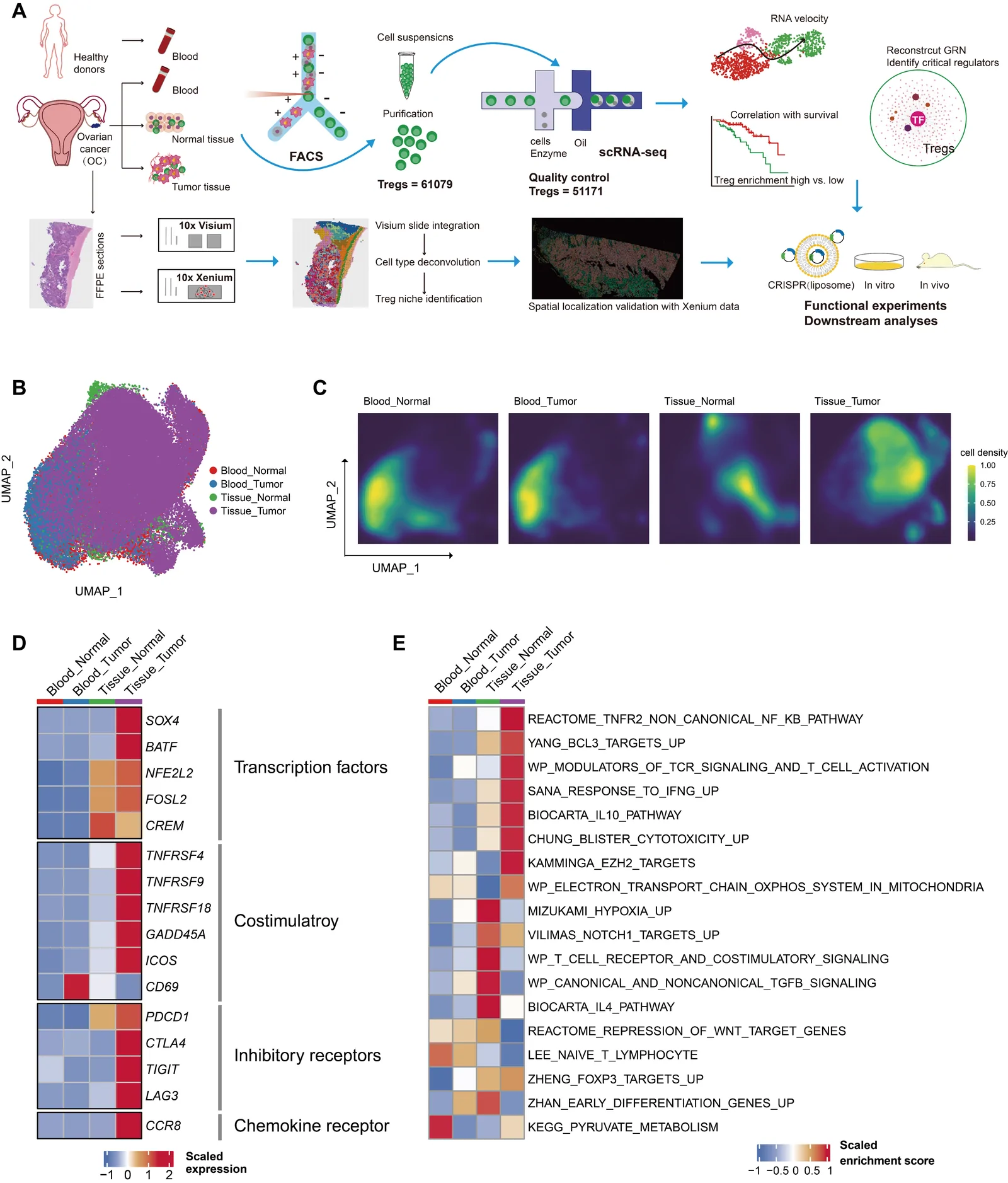
** Single-cell RNA sequencing delineates the subgroups of human ovarian cancer-associated Tregs. (A)** Single-cell RNA sequencing was conducted on 61,079 Tregs that were isolated by flow cytometry. These Tregs were sourced from peripheral blood samples obtained from healthy donors, peripheral blood of ovarian cancer patients, non-tumoral tissues, and tumoral tissues in ovarian cancer patients. The GEXSCOPE platform was utilized for this analysis. We further employed various bioinformatics approaches to identify cell types and states, infer differentiation drivers, and reconstructed Gene Regulatory Networks using SCENIC. **(B)** UMAP embedding and clustering of 51171 Tregs after removal of low-quality cells, color-coded according to source. **(C)** A two-dimensional galaxy plot of Tregs, grouped by cell source sample type, with lighter colors indicating higher cell density in the sample groups. **(D)** Heatmap of differentially expressed genes in ovarian cancer Tregs compared to all other cell populations. Expression levels are Z-score normalized across groups. **(E)** Heatmap showing the enrichment of selected gene sets across Treg subsets, stratified by tissue origin. Enrichment scores were calculated using the *AUCell* R package and row-scaled. KEGG, Kyoto Encyclopedia of Genes and Genomes.

In this study, peripheral blood mononuclear cells (PBMCs) from one healthy donor and one patient with OC, adjacent normal tissue from one OC patient, and tumor tissue from three cases of OC were collected. Using the gating strategy shown in **Figure S1A**, we isolated 61,079 viable CD4⁺CD25⁺CD127⁻ Tregs by fluorescence-activated cell sorting (FACS), which were subsequently subjected to single-cell RNA sequencing. Following rigorous bioinformatic quality control and filtering, transcriptional profiles from 51,171 high-quality Tregs were retained for downstream analysis, with each cell exhibiting an average detection of 1,833 genes. To complement the single-cell atlas with spatial architecture, we further integrated spatial transcriptomics with CRISPR-mediated perturbation experiments to investigate the regulatory programs shaping Treg phenotypes within the OC TME (**Figure 1A**).

The Shared Nearest Neighbor (SNN) algorithm was used to perform unsupervised clustering and displayed by UMAP, showing the distribution patterns of Tregs in four different sample origins (**Figure 1B**). **Figure 1C** displays the relative cellular densities of across above different sample groups. Importantly, Tregs from healthy donor and OC patient peripheral blood samples clustered similarly, whereas Tregs generated from the tissue showed a far higher degree of transcriptional dispersion. This trend was particularly evident in OC tissues, indicating TI-Tregs showed the highest degree of heterogeneity. Compared with circulating Tregs from peripheral blood, tissue-derived Tregs exhibited markedly elevated expression of the TFs *SOX4* and *BATF*, together with the phenotypic marker *PDCD1* (**Figure 1D**). This expression pattern suggests that Tregs within the tissue microenvironment may adopt a more activated immunoregulatory state, potentially contributing to strengthened local immune tolerance. Additionally, OC TI-Tregs exhibited notable upregulation of several activation markers, including TNFRSF4, TNFRSF9, TNFRSF18, GADD45A, and ICOS. This upregulation may be a biological response to activation signals within TME and could facilitate the immunosuppressive functions of Tregs.

To further elucidate the molecular characteristics of OC TI-Tregs, analysis of differentially expressed gene sets revealed an enrichment of genes associated with the atypical TNFR2-NF-κB signaling pathway 24, 25 in TI-Treg populations (**Figure 1E**). This was not only evident in the upregulation of the IL-10 signaling pathway but also in the strong response to interferon-γ (IFNG) signaling 26-28 and enhanced expression of genes such as *BCL3* and *EZH2*. These findings may reflect the regulatory role of TI-Tregs in modulating the response of CD8^+^ T cells to tumor cell attack. These analyses provided an overview of the expression signatures differentially enriched in OC TI-Tregs compared with Tregs from nontumor tissues and peripheral blood.

### Identification of tumor-specific TI-Treg subsets in ovarian cancer

**Figure 2:**
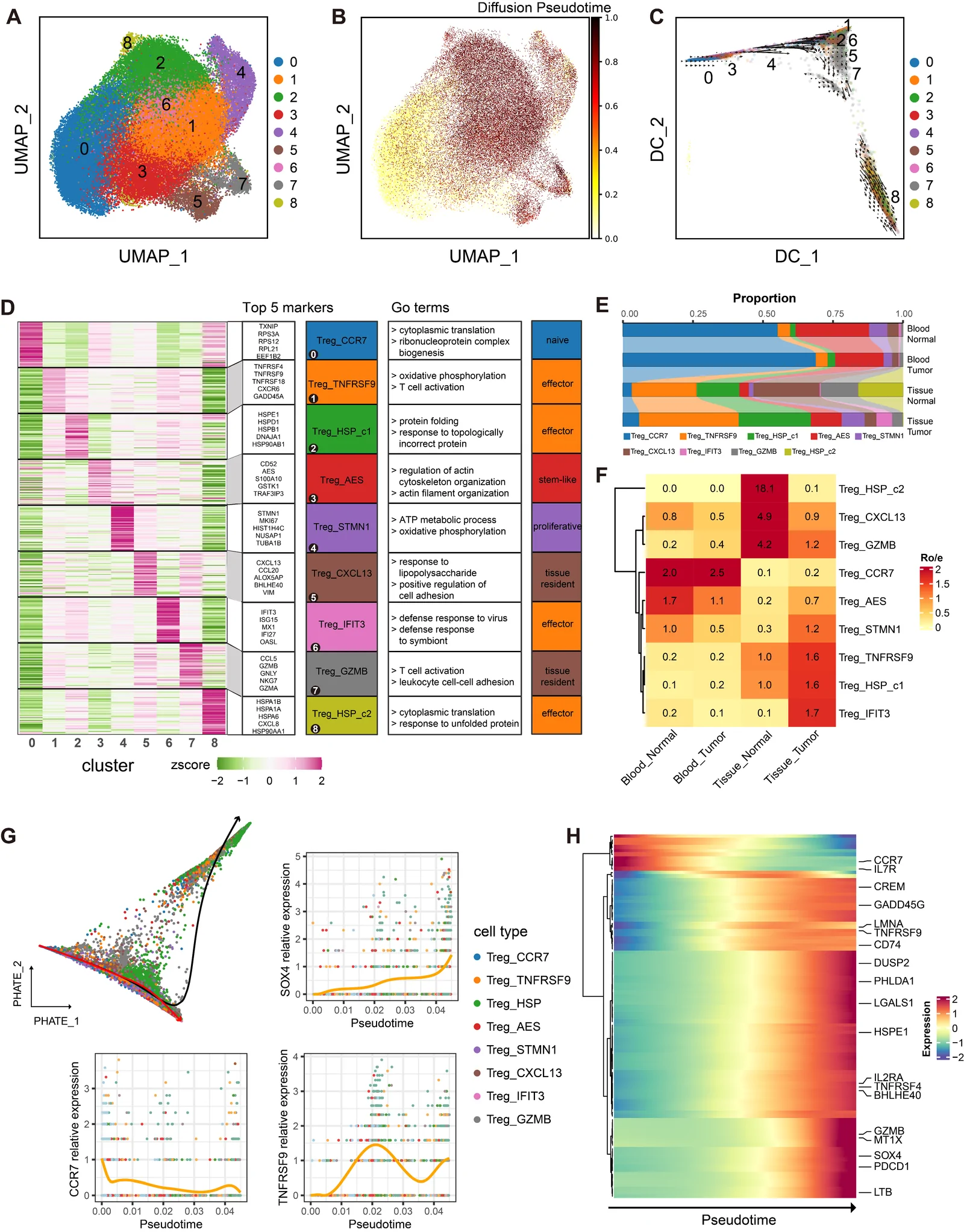
** Role of differentiated Tregs in ovarian cancer microenvironment. (A)** UMAP embedding of Tregs from all samples. Nine clusters were identified through unbiased clustering based on the Louvian graph. **(B)** UMAP colored by diffusion pseudotime progression (0.0, yellow, root; 1.0, dark red, terminal state), indicating the predicted developmental trajectory of Tregs. **(C)** Tregs labeled according to cell states and visualized in diffusion map embedding. **(D)** Heatmap displaying the top 50 significantly upregulated marker genes (FDR < 0.05) across clusters, alongside selected enriched Gene Ontology (GO) terms and their classification into five functional subsets. Expression levels are Z-score normalized. **(E)** Stacked bar plot illustrating the composition and relative proportions of the nine Treg clusters across different tissue and blood sample groups. **(F)** Heatmap displaying the tissue preference of each Treg cluster quantified by the ratio of observed to expected cell numbers (Ro/e). **(G)** Slingshot pseudotime trajectory of Tregs visualized on a PHATE plot, with line plots showing the dynamic expression of *SOX4*, *CCR7*, and *TNFRSF9* along pseudotime. Each dot represents an individual cell. **(H)** Heatmap showing the expression cascades of the top 100 dynamically regulated genes along the pseudotime trajectory.

Tregs from four sample types were classified into nine distinct cell clusters using a resolution of 0.5 (**Figure 2A**): Treg_CCR7, Treg_TNFRSF9, Treg_HSP_c1, Treg_AES, Treg_STMN1, Treg_CXCL13, Treg_IFIT3, Treg_GZMB, and Treg_HSP_c2. Pseudotime analysis revealed a differentiation trajectory of Treg clusters, with the three TI-Treg clusters located at the terminal differentiation endpoints (**Figure 2B-C**) 29. These clusters were further categorized into five functional subsets: the CCR7 cluster represented naïve Tregs, the AES cluster corresponded to stem-like Tregs, the STMN1 cluster represented proliferative Tregs, clusters TNFRSF9, HSP_c1, HSP_c2, and IFIT3 were combined as TI-Tregs, and CXCL13 and GZMB clusters were classified as tissue-resident Tregs (**Figure 2D**). The TNFRSF9, HSP, and IFIT3 clusters were significantly enriched in the OC TME, exhibiting tumor-specific and overlapping phenotypes compared to non-tumorous tissues (**Figure 2E-F**). Along pseudotime expression dynamics, genes marking naïve subsets, such as CCR7 and IL7R, exhibited a decreasing trend, whereas TI-Treg-associated TFs, including CREM and SOX4, showed an increasing expression pattern (**Figure 2G-H**). TNFRSF9/HSP/IFIT3 axis as a disease-relevant signature—and potential point of intervention—of TI-Treg biology in OC.

### Spatial distribution and clinical relevance of TI-Treg subsets in ovarian cancer

**Figure 3:**
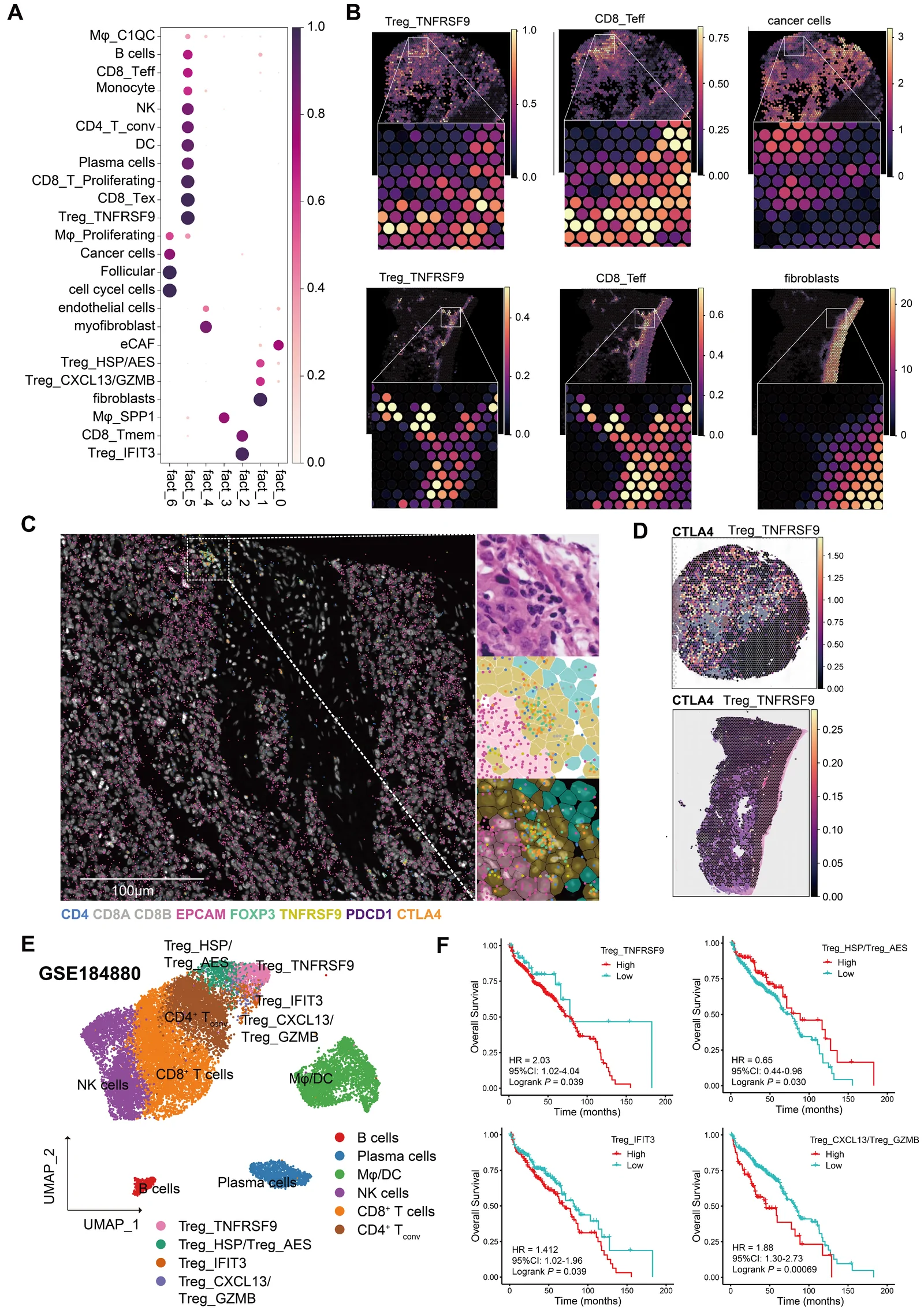
** Spatial architecture and clinical prognostic relevance of Treg subsets in ovarian cancer. (A)** Dot plot of estimated Non-negative Matrix Factorization (NMF) weights (columns) across various cell types (rows) from Visium spatial transcriptomics of two ovarian cancer tissues, profiling specific microenvironmental compartments. **(B)** Spatial deconvolution maps showing the estimated abundance and co-localization of the Treg_TNFRSF9 subset with CD8⁺ effector T cells, cancer cells, and fibroblasts in two representative ovarian cancer tissues. Boxed areas are magnified in the insets below. **(C)** Multiplexed *in situ* visualization of tumor and immune cell markers (CD4, CD8A/B, EPCAM, FOXP3, TNFRSF9, PDCD1, and CTLA4) using Xenium technology (left). Right panels (top to bottom) present corresponding H&E staining, cell type segmentation masks, and high-resolution spatial localization within the designated region. **(D)** Spatial expression patterns of *CTLA4* in the Treg_TNFRSF9 cluster across two ovarian cancer tissue sections. **(E)** UMAP visualization and cell-type clustering of 59,324 single cells from the validation dataset (GSE184880), with Treg subclusters identified using the top 200 differentially expressed genes from this study. **(F)** Kaplan–Meier survival curves illustrating the association between the abundance of distinct Treg subsets and overall survival in ovarian cancer patients. Hazard ratios (HR) and *P*-values were determined by univariate Cox regression and log-rank tests, respectively.

To characterize the spatial distribution of Treg subpopulations, we performed cell type deconvo-lution analysis on Visium spatial transcriptomics data derived from two OC samples, and mapped a scRNA-seq atlas of 24 reference cell types from seven cases of high-grade serous OC and five adjacent non-tumor tissues composed of 59,324 cells (**Figure S2A-D**). We selected the top 200 differentially expressed genes from our OC-related Treg subsets as an enrichment gene set to score the clusters in GSE184880, categorizing 2,056 Tregs into four clusters: Treg_TNFRSF9, Treg_HSP/AES, Treg_IFIT3, and Treg_CXCL13/GZMB. The analysis revealed that the Treg_TNFRSF9 cluster, showed prominent expression profiles in tumor immune cell-rich regions (**Figure 3A**). Further investigation revealed the spatial co-localization of the Treg_TNFRSF9 cluster with effector CD8⁺T cells (**Figure 3B-C**), suggesting that TI-Tregs potentially exert immunosuppressive functions through direct cell-cell interactions or secretion of suppressive cytokines. Additionally, spatial transcriptomic analysis revealed that the Treg_TNFRSF9 cluster was predominantly enriched near the tumor boundary (**Figure S2E-F**), with markedly elevated expression of immune checkpoint molecule CTLA-4 (**Figure 3D**). We further analyzed the correlation between patient survival and the enrichment of these Treg subsets, alongside deconvoluted clusters of CD8⁺ T cells, NK cells, B cells, and macrophages/dendritic cells within the TCGA_OV cohort (**Figure 3E**).

As shown in** Figure 3F**, univariate Cox proportional hazards regression analysis revealed that Treg_TNFRSF9 subset was significantly associated with unfavorable survival outcomes (HR = 2.03, 95% CI 1.02-4.04). In contrast, the Treg_HSP/AES subset showed a possible protective role with comparatively reduced HR of 0.65 (95% CI 0.44-0.96). The Treg_IFIT3 and Treg_CXCL13/GZMB populations were also related to lower survival probability, with HR values of 1.412 and 1.88, respectively.

### Integrated single-cell and functional analyses reveal SOX4 as a key transcription factor in ovarian cancer TI-Tregs

**Figure 4:**
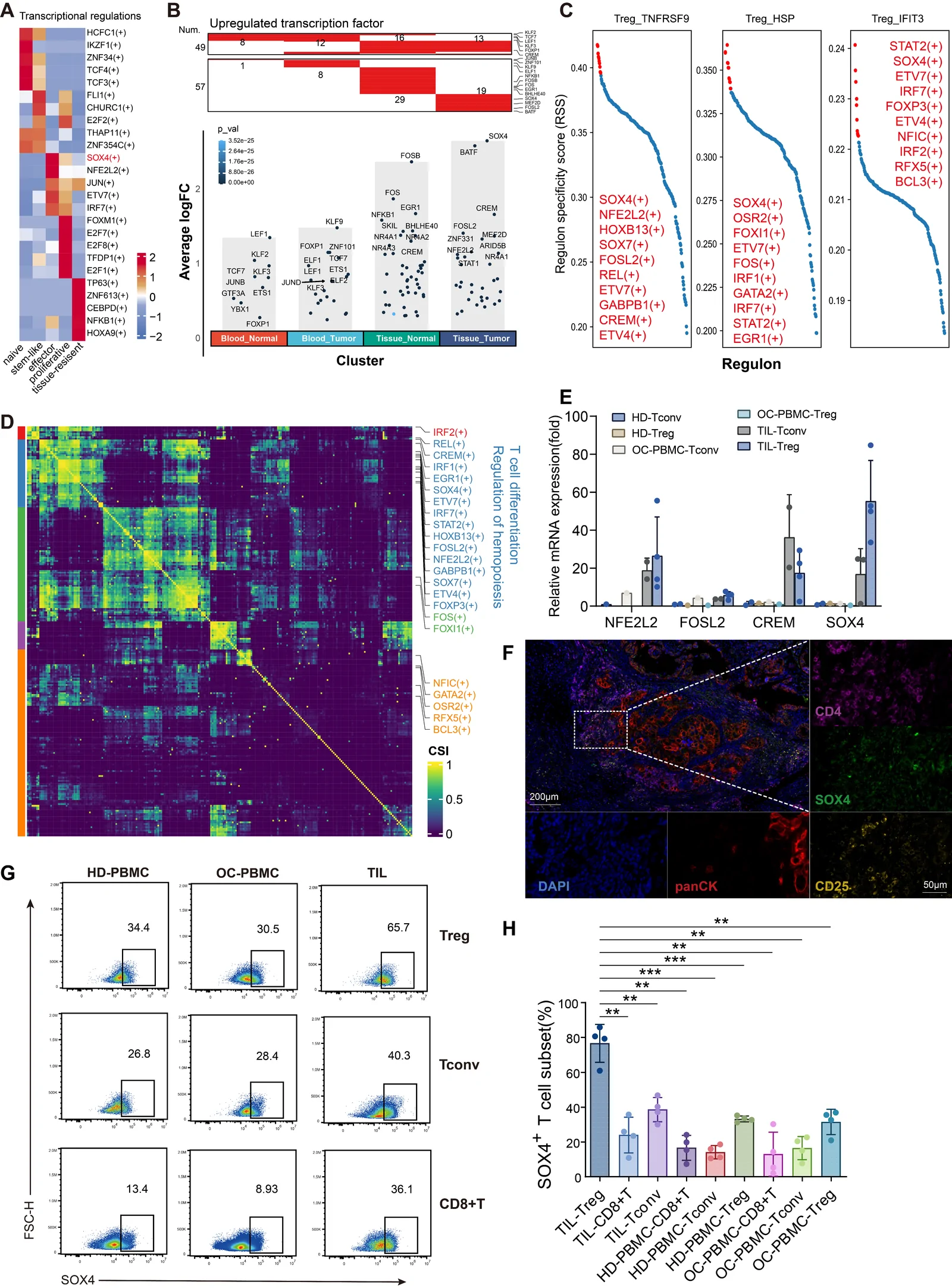
** Identification and validation of SOX4 as a master transcription factor defining ovarian cancer TI-Treg identity. (A)** Heatmap of SCENIC analysis displaying the row-scaled regulon activity scores (estimated via AUCell) of top-ranked transcription factors across five functional Treg subsets. SOX4(+) is highlighted as a dominant regulon in effector TI-Tregs. **(B)** Distribution of differentially expressed transcription factors (TFs) across the four sample groups. The upper panel indicates TFs shared by at least two groups, with numbers denoting TF counts; the lower panel displays group-specific TFs. **(C)** Regulon Specificity Score (RSS) ranking for individual TI-Treg clusters (Treg_TNFRSF9, Treg_HSP, and Treg_IFIT3), highlighting top candidate regulons. **(D)** Hierarchical clustering of the Connection Specificity Index (CSI) matrix revealing co-regulated transcription factor modules. **(E)** Relative mRNA expression of candidate TFs (NFE2L2, FOSL2, CREM, and SOX4) across T cell subsets isolated from healthy donors (HD), OC peripheral blood (OC-PBMC), and tumor-infiltrating lymphocytes (TIL). Data are normalized to NFE2L2 levels in HD-Tconv cells. Each dot represents a biological replicate; bars indicate mean ± SD. **(F)** Multiplex immunohistochemistry of ovarian cancer tissue sections showing the spatial distribution of SOX4 (green), CD4 (magenta), CD25 (yellow), and panCK (red). DAPI (blue) was used for nuclear staining. Insets show the presence of SOX4⁺ Tregs in ovarian cancer. **(G, H)** Representative flow cytometry plots **(G)** and statistical quantification **(H)** of SOX4⁺ cell frequencies within Treg, Tconv, and CD8⁺ T cell populations from HD-PBMC, OC-PBMC, and TIL samples. Data are presented as mean ± SD; *P < 0.05; **P < 0.01; ***P < 0.001; ns, not significant.

The phenotype during T cell activation are dynamically governed by TFs and their associated cofactors, which work together to determine cell function. The TF regulatory networks of the five functional Treg subsets were inferred with SCENIC analysis 30. In effector Tregs subsets, enrichment of regulons of SOX4 and NFE2L2 was increased (**Figure 4A**). Consistent with the findings from “FindAllMarkers” analysis, which identified SOX4, BATF, CREM and FOSL2 as highly upregulated TFs in OC tissues (**Figure 4B**). Among these candidates, SOX4 demonstrated the highest Regulon Specificity Score (RSS) across multiple TI-Treg populations, including Treg_TNFRSF9 and Treg_HSP (**Figure 4C**). Regulon activity scores (RAS) were systematically compared for all regulon pairs using the Connection Specificity Index (CSI). Specifically, SOX4, FOSL2, CREM, and NFE2L2 were clustered together in a highly specific common regulatory module associated with T-cell differentiation and hematopoiesis. (**Figure 4D**). Subsequent qRT-PCR validation further confirmed that SOX4 showed the highest expression level in the TI-Treg subset compared with the other candidate TFs (**Figure 4E**). To further validate the protein-level existence and spatial localization of the SOX4 driven TI-Treg subpopulation, multiplex immunohistochemistry (mIHC) staining was performed (**Figure 4F**). By co-staining for CD4, CD25, and SOX4, we successfully identified a population of SOX4⁺ Tregs infiltrating the tumor nests and stroma. Moreover, flow cytometric analysis confirmed that SOX4⁺ Tregs were highly enriched among tumor-infiltrating lymphocytes compared to Tcon and CD8⁺ T cells, as well as their peripheral blood counterparts (**Figure 4G-H**). Collectively, these findings establish SOX4 as a lineage specifying TF that determines TI-Tregs flexibility in the OC microenvironment.

**Figure 5:**
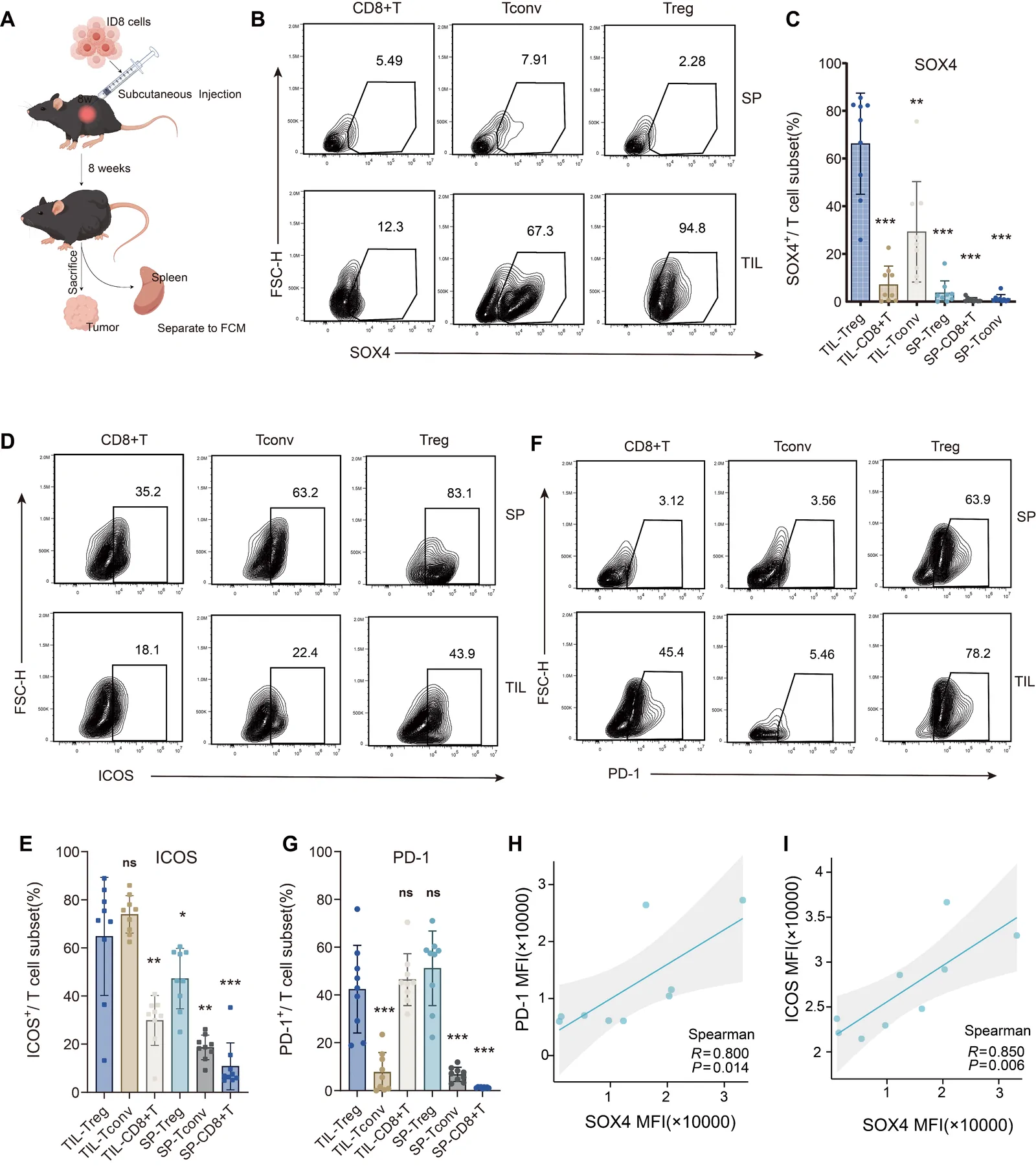
** Elevated SOX4 expression correlates with activation and checkpoint marker profiles in a murine ovarian cancer model. (A)** Schematic representation of the ID8-derived subcutaneous ovarian cancer model establishment and experimental workflow in C57BL/6 mice. **(B-C)** Representative flow cytometry plots **(B)** and statistical quantification **(C)** of SOX4⁺ cell frequencies within CD8⁺ T, Tconv, and Treg populations isolated from tumor-infiltrating lymphocytes (TILs) and spleens (SP). **(D-E)** Representative flow cytometry plots** (D)** and corresponding quantification **(E)** of ICOS⁺ cell frequencies across the indicated T cell subsets in TIL and SP compartments. **(F-G)** Representative flow cytometry plots **(F)** and corresponding quantification **(G)** of PD-1⁺ cell frequencies across the indicated T cell subsets. **(H-I)** Spearman correlation analysis between SOX4 mean fluorescence intensity (MFI) and PD-1 MFI** (H)** or ICOS MFI **(I)** in murine TI-Tregs. Data in bar graphs are presented as mean ± SD. *P < 0.05, **P < 0.01, ***P < 0.001, ns, not significant.

To further elucidate the importance of SOX4, we established a subcutaneous OC model in C57BL/6 mice (**Figure 5A**). Consistently, SOX4 expression was significantly higher in murine TI-Tregs than in other tumor-infiltrating T-cell subsets or splenic T cells (**Figure S1B, 5B-C**). Phenotypically, SOX4⁺ Tregs maintained a highly activated yet strongly suppressive phenotype, characterized by elevated expression of ICOS and PD-1(**Figure 5D-G**). Moreover, in murine OC–infiltrating Tregs, SOX4 levels correlated strongly with the activation marker ICOS and the inhibitory receptor PD-1 (**Figure 5H-I**). In summary, our integrated bioinformatic and experimental evidence identify SOX4 as a hallmark of human and murine OC TI-Tregs, highlighting its essential role in sustaining their functional activity and immunosuppressive program.

### SOX4 regulates phenotypic marker expression in TI-Tregs

**Figure 6:**
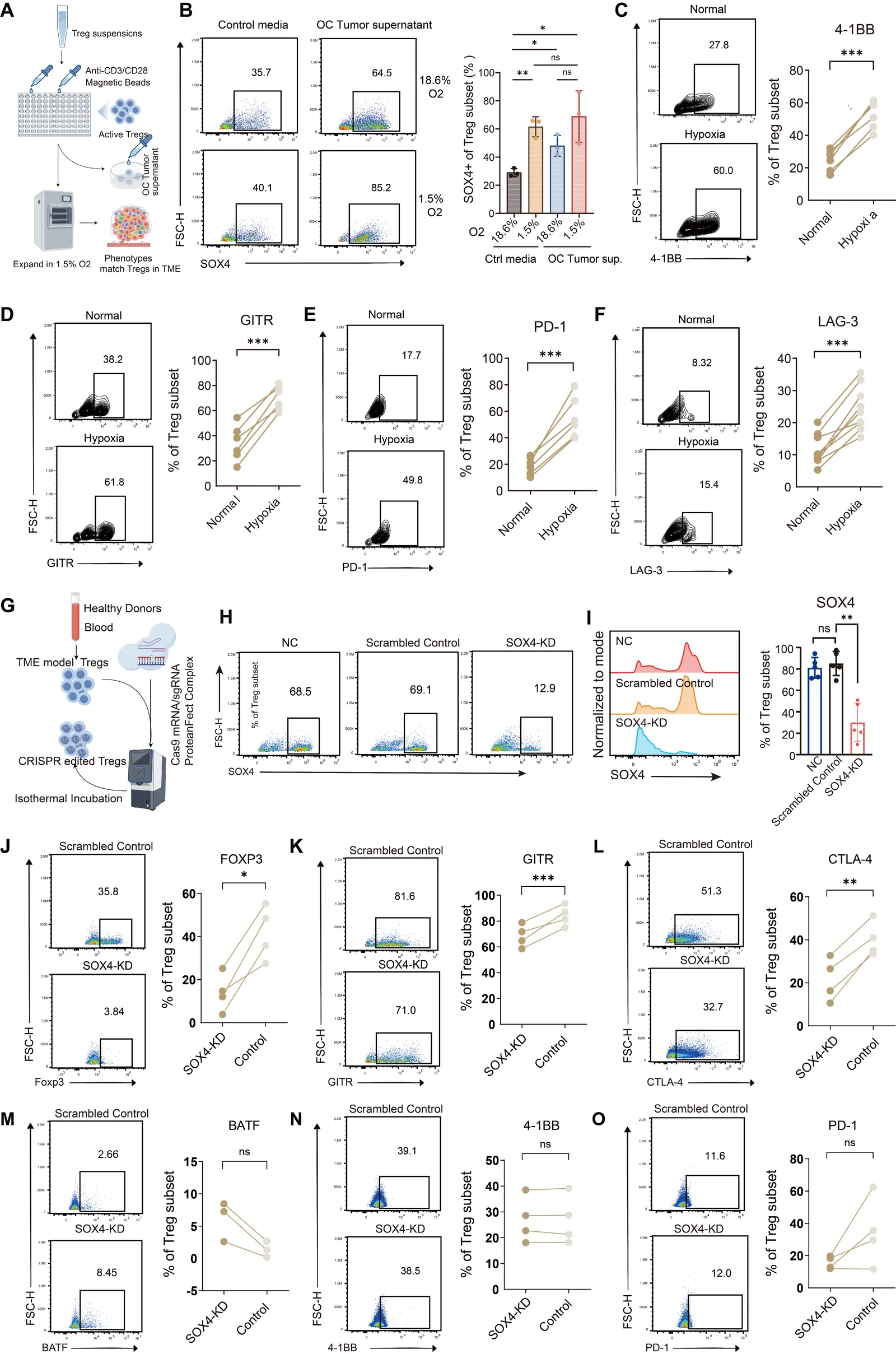
** SOX4 regulates the effector phenotype and functional stability of TI-Tregs. (A)** Experimental schematic illustrating the activation and culture of primary human Tregs in patient-derived tumor-conditioned medium under hypoxia (1.5% O_2_). **(B)** Representative flow-cytometry plots and summary statistics demonstrating that SOX4 expression in primary human Tregs is modulated by simulated tumor microenvironment conditions. Cells were grown either in standard medium or 50 % ovarian-cancer conditioned medium and maintained under normoxia (18.6% O₂) or hypoxia (1.5% O₂); hypoxic CM culture produced the highest SOX4 levels. **(C-F)** Flow cytometric assessment showing that hypoxia significantly upregulates the expression of activation markers 4-1BB (C) and GITR (D), as well as inhibitory receptors PD-1 (E) and LAG-3 (F). **(G)** Schematic workflow for CRISPR/Cas9-mediated SOX4 knockdown in Tregs. **(H)** Representative flow cytometry plots demonstrating the efficiency of SOX4 knockdown (SOX4-KD) in Tregs. **(I)** Histogram overlays showing SOX4 fluorescence intensity in the NC (red), Scrambled Control (orange), and SOX4-KD (blue) groups. Statistical quantification of the percentage of SOX4⁺ Tregs across biological replicates. **(J-L)** Flow cytometric analysis revealing that SOX4 depletion leads to a significant downregulation of core Treg functional markers, including FOXP3, GITR, and CTLA-4. **(M-O)** Representative plots showing that the expression of BATF, 4-1BB, and PD-1 remains largely unaffected following SOX4 knockdown in this context. Data are presented as median values ± SD; each dot represents an independent biological replicate. *P < 0.05; **P < 0.01; ***P < 0.001; ns, not significant.

To investigate the regulatory role of SOX4 in TI-Treg specialization, we developed an *in vitro* system to simulate the OC TME. Primary human Tregs were cultured in patient-derived tumor-conditioned medium under hypoxic conditions (1.5% O₂) (**Figure 6A**). Flow cytometry analysis showed that exposure to OC tumor supernatant under hypoxic conditions markedly enhanced SOX4 expression in Tregs compared with standard culture conditions (**Figure 6B**), resembling TI-Treg features with immuno-suppressive activity. Within this tumor micro-environment–mimicking *in vitro* system, Tregs acquired elevated expression of activation-associated markers, including 4-1BB and GITR, together with inhibitory checkpoint molecules such as PD-1 and LAG-3 (**Figure 6C–F**). Then, we applied CRISPR/Cas9-mediated gene editing to inhibit SOX4 expression in primary Tregs based on this preconditioned system for further investigation **(Figure 6G)**. Notably, we obtained approximately 81.3% SOX4 silencing efficiency at the mRNA level, with the expression decreased to 12.9% in the SOX4-KD group, compared with 68.5% and 69.1% in the negative control (NC) and scrambled control groups, respectively **(Figure 6H-I)**. Importantly, SOX4 depletion in Tregs significantly downregulated FOXP3, GITR, and CTLA-4** (Figure 6J–L)** while it did not affect BATF, 4-1BB, and PD-1 expression **(Figure 6M–O)**. These results indicate that SOX4 is a critical and specific regulator of the suppressive phenotype and functional stability of Tregs within the OC TME.

### SOX4 sustains TI-Treg function through oxidative phosphorylation–dependent regulation of FOXP3

**Figure 7:**
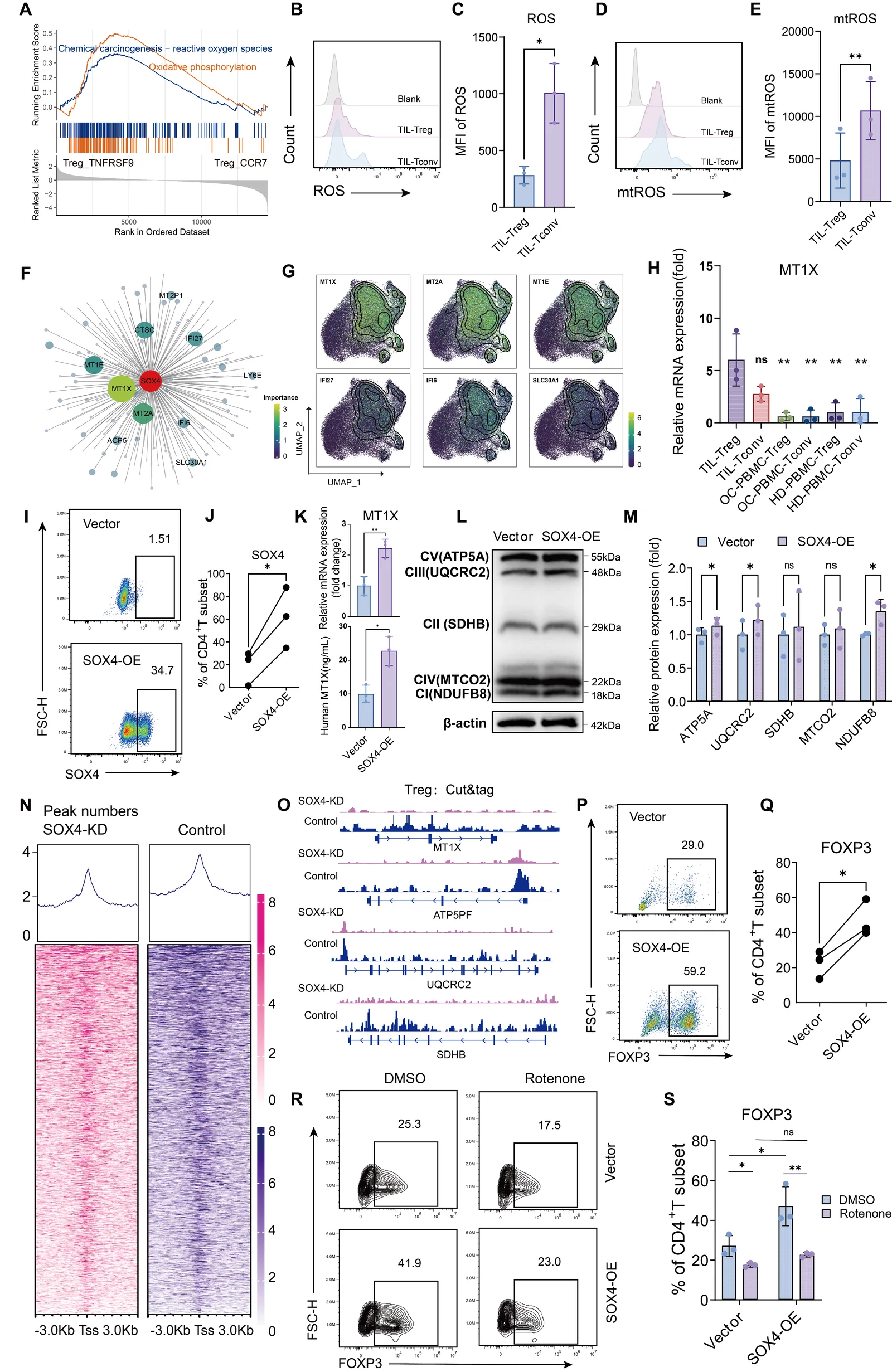
** SOX4 Regulates mitochondrial metabolism and FOXP3 expression to drive Treg functional programming. (A)** GSEA profiles reveal enriched pathways between Treg_TNFRSF9 and Treg_CCR7 clusters. **(B-E)** Representative flow cytometry histograms and statistical quantification of total intracellular ROS (B-C) and mitochondrial ROS (mtROS) (D-E) levels in TI-Tregs and TI-Tconvs, measured by mean fluorescence intensity (MFI). **(F)** Transcription factor regulatory network centered on SOX4 and its downstream target genes in TI-Tregs. Color gradients (yellow to purple) depict expression levels, while the contour map shows cell cluster density distributions. **(G)** UMAP embeddings illustrate the distribution of downstream target genes across Treg cell clusters. **(H)** qPCR validation of MT1X mRNA expression levels in T cell subsets from Tumor-infiltrating lymphocytes (TIL), ovarian cancer (OC), PBMC, and healthy control (HC). **(I–J)** Representative flow-cytometry plots (I) and quantification (J) for SOX4 overexpression in CD4⁺ T cells. **(K)** Relative mRNA expression of *MT1X* (top) and MT1X protein levels (bottom) measured by ELISA in Vector vs. SOX4-OE CD4⁺ T cells. **(L–M)** Western blot (L) representatives and densitometry (M) of mitochondrial respiratory-chain–related proteins in vector vs. SOX4-OE CD4⁺ T cells. **(N)** Heatmaps and average profile plots of SOX4 CUT&Tag signal enrichment around Transcription Start Sites (TSS) in SOX4-KD vs. Control cells. **(O)** Representative CUT&Tag tracks showing SOX4 binding occupancy and chromatin accessibility at the MT1X, ATP5PF, UQCRC2, and SDHB gene loci. **(P-Q)** Representative flow cytometry plots (P) and statistical quantification (Q) of FOXP3 expression in CD4⁺ T cells following SOX4 overexpression. **(R–S)** Representative flow cytometry plots (R) and statistical summary (S) of FOXP3 expression in Vector and SOX4-OE CD4⁺ T cells treated with Dimethyl sulfoxide (DMSO) or the OXPHOS inhibitor Rotenone. Horizontal lines indicate median values; error bars represent standard deviation (SD). *P < 0.05; **P < 0.01; ***P *<* 0.001; ns, not significant.

GSEA results revealed that high enrichment of oxidative phosphorylation (OXPHOS) pathways in the effector TI-Treg clusters (Treg_TNFRSF9 vs. Treg_CCR7) (**Figure 7A**). Fluorescent-probe assays showed that TI-Tregs had lower levels of intracellular and mitochondrial reactive oxygen species (ROS) that conventional T cells (Tconv), indicating that TI-Tregs have enhanced metabolic fitness that maintains high OXPHOS activity without compromising redox homeostasis (**Figure 7B–E**). To identify the upstream drivers of this metabolic program, we constructed a SOX4-centic regulatory network (**Figure 7F**). UMAP visualization of SOX4 downstream target genes revealed that MT1X, MT2A, MT1E, IFI27, IFI6, and SLC30A1 were preferentially enriched within specific OC TI-Treg clusters, among which MT1X displayed the strongest expression intensity (**Figure 7G**). This distribution pattern suggested a possible functional relevance of these genes in shaping TI-Treg phenotypes. Specifically, these data demonstrate that SOX4 may regulate MT1X to impact OXPHOS in OC TI-Tregs, leading to enhanced metabolic adaptability and effector functions. Furthermore, qPCR analysis confirmed MT1X expression was significantly elevated in TI-Tregs compared to PBMC-derived T cells and Tconvs (**Figure 7H**).

To further investigate downstream SOX4-dependent effects, we ectopically overexpressed SOX4 in CD4⁺ T cells and increased transduction efficiency from 1.51% to 34.7%, as confirmed by flow cytometry (**Figure S1C, 7I -J**). Additional analyses showed that SOX4 overexpression significantly increased MT1X expression at the transcript and protein level. (**Figure 7K**). In parallel, elevated SOX4 expression was associated with increased abundance of several core respiratory-chain components involved in OXPHOS, including ATP5A, UQCRC2, and NDUFB8 (**Figure 7L-M**). Mechanistically, CUT&Tag profiling showed that loss of SOX4 decreased chromatin accessibility at the MT1X locus as well as multiple OXPHOS-associated genomic regions (**Figure 7N-O**), suggesting these genes directly regulated by SOX4. Besides, Clustering analysis of CUT&Tag and ATAC-seq data identified substantial differences in chromatin accessibility across key mitochondrial OXPHOS loci between OC tissues and healthy peripheral blood samples, with tumor-derived cells exhibiting stronger and more extensive accessibility peaks (**Figure S3A–D**).

Given that SOX4 knockdown markedly decreased FOXP3 expression in Tregs, we next explored whether SOX4 influences CD4⁺ T cell differentiation toward a Treg-like state. In agreement with this hypothesis, the expression of FOXP3 is highly associated with SOX4 activity (**Figure 7P-Q**). Functional metabolic assays further demonstrated that SOX4 overexpression increased intracellular ATP production without significantly altering total ROS or mitochondrial ROS levels (**Figure S3E–J**). In addition, secretion of the cytokines IL-10 and TGF-β was markedly elevated after SOX4 overexpression (**Figure S4**), further supporting its role in promoting Treg-like differentiation and immunoregulatory function. Pharmacological inhibition of OXPHOS by rotenone abolished the restoration of FOXP3 expression even in SOX4-overexpressing cells (**Figure 7R-S**), indicating that SOX4-mediated FOXP3 induction depends on intact mitochondrial oxidative phosphorylation. Taken together, these results identify SOX4 as a critical transcriptional metabolic regulator in OC TI-Tregs, which facilitates MT1X-OXPHOS reprogramming to FOXP3 maintenance and immunosuppressive function.

### Synergistic effect of Anti-PD-1 and rotenone in inhibiting tumor progression via modulating Treg phenotypes

**Figure 8:**
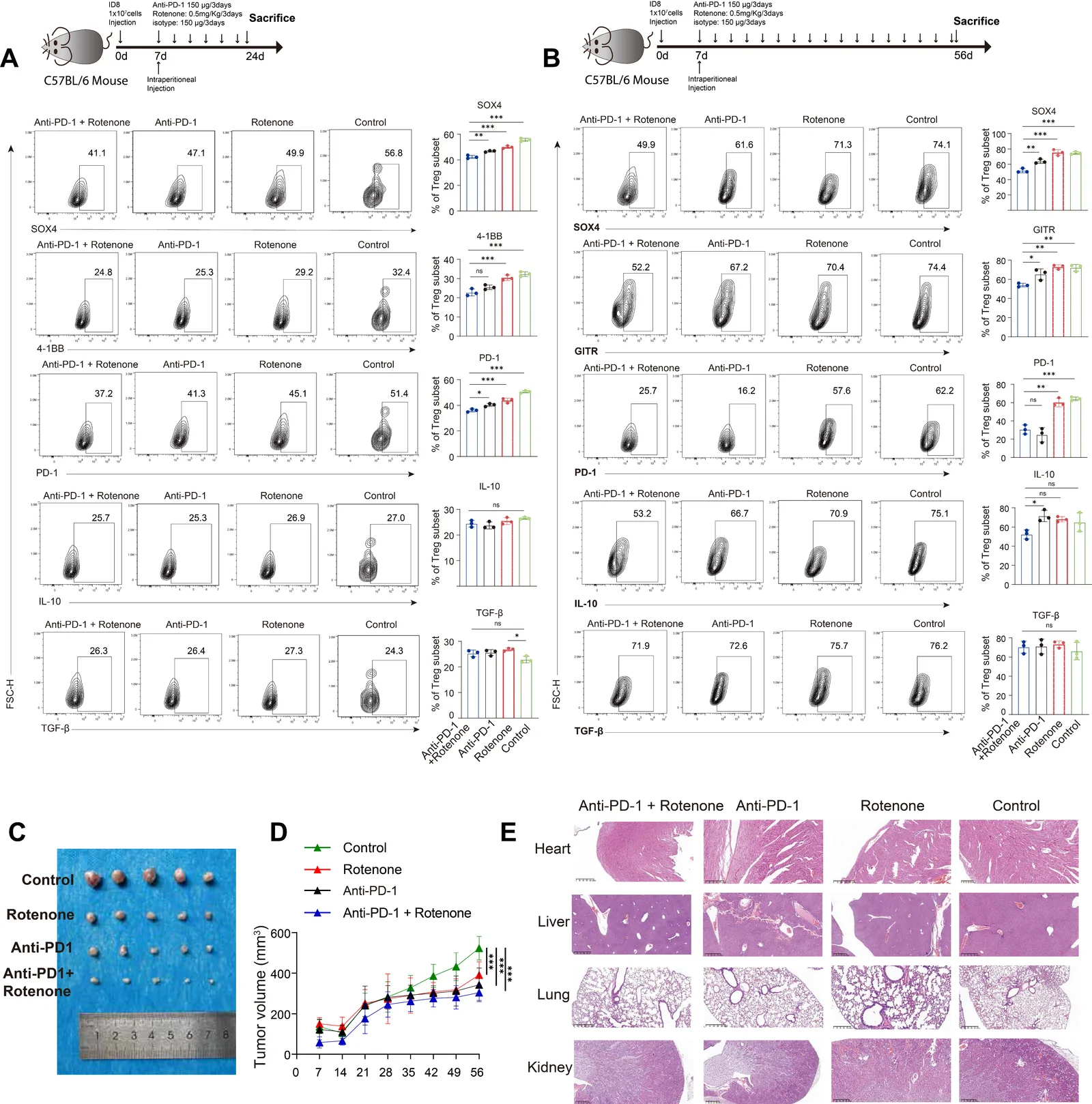
** Combined anti-PD-1 and rotenone treatment inhibits ovarian cancer progression by modulating the effector phenotypes of TI-Tregs. (A)** Phenotypic analysis of tumor-infiltrating Treg (TI-Tregs) in the early stage of ovarian cancer (3.5 weeks). Flow cytometric analysis showing a significant reduction in the proportion of SOX4⁺ Tregs within the TI-Treg population in the combination group (Anti-PD-1 + Rotenone) compared to the control. The expression of the inhibitory molecule PD-1 and the activation marker 4-1BB on TI-Tregs was significantly downregulated, whereas no significant changes were observed in the secretion of cytokines (IL-10, TGF-β). **(B)** Phenotypic evolution of TI-Tregs in the terminal stage of ovarian cancer (8 weeks). During the progression from early to late stages, the proportion of SOX4⁺ Tregs and the expression of phenotypic markers increased. Combination therapy significantly suppressed the expansion of the SOX4⁺ subpopulation and reduced PD-1 and GITR expression more effectively than in the early stage. Cytokine levels remained comparable across all groups. **(C)** Gross morphology of subcutaneous tumors. Representative images of tumors harvested from the four experimental groups. The combination treatment group exhibited significantly smaller tumor sizes compared to the control and monotherapy groups. **(D)** Tumor growth curves. Quantitative monitoring of tumor volumes over 56 days showing that the combination of Anti-PD-1 and Rotenone leads to the most potent inhibition of tumor growth (***P < 0.001).** (E)** H&E staining of major organs. Histological assessment of the heart, liver, lung, and kidney showed no significant pathological changes, metastatic lesions, or severe inflammation, demonstrating the high safety profile and low systemic toxicity of the combination therapy.

We next investigated whether the OXPHOS inhibitor rotenone could modulate tumorigenesis and improve the therapeutic efficacy of PD-1 blockade *in vivo*, further exploring the translational potential of our findings. At the early stage of tumor development (3.5 weeks;** Figure 8A and Figure S5A**), flow cytometric analysis showed that the proportion of SOX4⁺ Tregs was reduced in C57BL/6 mice receiving combination therapy compared with controls. In parallel, expression levels of the inhibitory receptor PD-1 and the activation-associated marker 4-1BB on TI-Tregs were also decreased following treatment. By contrast, secretion of suppressive cytokines, including IL-10 and TGF-β, did not differ substantially at this stage. By the terminal stage of tumor progression (8 weeks; **Figure 8B**), the inhibitory effect of the combined therapy (Anti-PD-1+rotenone) on SOX4⁺ TI-Tregs became more evident. Consistent with results from the early phase, the expression of PD-1 and GITR remained significantly lower in treated mice. To evaluate whether these effects extended beyond the local tumor microenvironment, systemic immune responses were further evaluated in peripheral lymphoid tissues. Flow cytometry analysis of tumor-draining lymph nodes and spleens from tumor-bearing mice demonstrated that anti-PD-1 plus rotenone therapy increased the proportion of CD8⁺ T cells, whereas the overall CD4⁺ T-cell population remained relatively stable (**Figure S5B–C**). These findings suggest that the combined therapy may simultaneously alleviate local immunosuppression while promoting CD8⁺ T cell–mediated systemic antitumor immunity. The biological consequences of the immunological changes were also reflected in tumor burden and growth dynamics. The mean tumor volume was significantly reduced in mice receiving the combined treatment compared with control and monotherapy groups (**Figure 8C-D**), supporting a synergistic antitumor effect of anti-PD-1 and rotenone.

Histopathological examination of major organs, including the heart, liver, lung, and kidney, revealed no detectable metastatic lesions or obvious inflammatory injury in the combination-treatment group (**Figure 8E**). In addition, endpoint body weight measurements together with serum biochemical indicators, including Creatinine (CREA), Alanine Aminotransferase (ALT), Aspartate Aminotransferase (AST), and Total Bilirubin (TBIL), showed no evidence of overt systemic toxicity (**Figure S5D**). Collectively, these data indicate that combined anti-PD-1 and rotenone therapy demonstrates significant anticancer efficacy with a favorable safety profile in OC.

## Discussion

Immune cell infiltration wihtin TME plays a pivotal role in driving tumor progression and impacting clinical outcomes. Although comprehensive profiling of tumor-infiltrating immune cells (TIICs) holds great potential to unveil cryptic mechanisms of immune escape and inspire novel therapeutic strategies, the pronounced heterogeneity and dynamic nature of these intra-tumoral infiltrates pose a major obstacle to precision oncology. This cellular complexity is evident in OC, where Tregs are characterized by a highly activated phenotype with superior suppressive capacity, reinforcing local immunotolerance through a multifaceted programmatic wiring that involves the secretion of immunosuppressive cytokines (TGF-β and IL-10), upregulation of inhibitory checkpoints (PD-1 and CTLA-4), local IL-2 deprivation, and metabolic rewiring 31, 32. Mollaoglu *et al*. reported that OC cells serve as a primary source of IL-4, a key cytokine that drives resistance to anti-PD-1 therapy by directing the short-range formation of immunosuppressive, macrophage-controlled neighborhoods 33. Nevertheless, how these diverse immunosuppressive programs are stratified across discrete, functionally specialized Treg subpopulations, and the precise transcriptional trajectories driving their polarization within the OC TME, remain poorly resolved. Reassuringly, recent technological advances in high-throughput scRNA-seq and spatial transcriptomics have enabled unprecedented deconstruction of such complex tumor ecosystems 34. Driven by the need to fill this critical knowledge gap, we applied this advanced framework to map the single-cell landscape of OC associated TI-Tregs.

Our study provides a comprehensive scRNA-seq atlas of Tregs derived from healthy individuals and patients with OC, spanning peripheral blood, peri-tumoral regions, and tumor tissues. This multi-compartment profiling reveals clear transcriptomic divergence among Tregs from four distinct sample types. Comparative analyses demonstrate pronounced differences between tissue-resident and peripheral blood Tregs, with tumor-infiltrating populations exhibiting the greatest degree of transcriptional heterogeneity. In particular, key TFs (SOX4 and BATF) and functional markers (OX40 and PDCD1), were consistently elevated in tissue-derived Tregs relative to circulating counterparts, suggesting active remodeling of Treg states within the tissue microenvironment and a concomitant reinforcement of local immune tolerance. Moreover, OC associated TI-Tregs displayed broad upregulation of activation-associated molecules, such as TNFRSF4, TNFRSF9, TNFRSF18, GADD45A, and ICOS. This expression pattern likely reflects sustained stimulation within the TME and may contribute to the reinforcement of their immunosuppressive capacity.

In adjacent ovarian tissues, enrichment of hypoxia, NOTCH1, TGF-β, and IL-4 related signaling pathways suggests that Tregs may reside in a relatively plastic and transcriptionally primed state, potentially retaining proliferative potential. In contrast, the marked suppression of WNT-associated gene programs in tumor-infiltrating Tregs implies a shift toward a more terminally differentiated and functionally specialized phenotype within the TME. Interestingly, peripheral blood Tregs from healthy individuals exhibited selective enrichment of pyruvate metabolism pathways, which may reflect increased energetic demands associated with active cellular maintenance and turnover. Taken together, our scRNA-seq analysis reveals substantial phenotypic heterogeneity among Tregs across OC–related compartments, accompanied by distinct transcript-tional and metabolic programs. In particular, the coordinated upregulation of TFs such as SOX4 and BATF, together with enhanced oxidative phosphorylation–related activity, underscores the emergence of a specialized TI-Treg state that may play a key role in shaping the immunosuppressive TME.

To elucidate the molecular characteristics of OC TI-Tregs, differential gene expression analysis reveals enrichment of gene sets associated with the TNFR2-NF-κB signaling pathway. This is manifested not only by upregulation of the IL-10 signaling pathway but also by a strong response to IFNG signaling and increased expression of genes such as BCL3 and EZH2. These findings may reflect the regulatory role of OC TI-Tregs in responding to CD8^+^ T cell attacks on tumor cells. In the TME, TI-Tregs suppress the effector function of CD8^+^ T cells through local proliferation and modulate the expression of apoptosis-related mechanisms to prevent the overactivation of systemic immunosuppressive processes. The unique and heterogeneous nature of the TME may promote environments rich in specific cytokines that foster Treg proliferation while also activating signals that drive apoptosis.

Further pseudotime trajectory analysis of Treg cell clusters aligns with their functional states. Treg subgroups gradually recruit and differentiate from a naive peripheral state to an effector state in the TME, performing immunosuppressive functions, with other subgroups such as tissue-resident and proliferative types preceding effector differentiation. This highlights the developmental process of regulatory activation of Treg function within the TME. Recent studies suggest that only a small portion (< 10%) of Treg-associated genes are directly regulated by FOXP3 binding, indicating that relying solely on FOXP3 is insufficient to control the entire differentiation process of Tregs or to fully endow mature Tregs with complete functionality 35. This suggests that other TFs act upstream, downstream, or concurrently with FOXP3 to co-regulate Treg characteristic functions and differentiation pathways. In our study, differential expression of TFs among Treg subgroups partly explains heterogeneity, regulating the migration, survival, and functional attributes of Treg subgroups. Our findings reveal that in OC TI-Tregs, SOX4 exhibits the highest gene expression levels among all TFs and is significantly enriched in the effector TI-Treg subgroup. SOX4 is a TF and a member of the SOX (SRY-related HMG-box) family, known to be upregulated in various cancers, including breast, prostate, lung, colorectal, and pancreatic cancers. Recent findings indicate that SOX4^+^ naive T cells significantly contribute to thymic migration within the human immune system 36. SOX4 promotes tumor progression by regulating genes involved in epithelial-mesenchymal transition (EMT) 37, and its overexpression is often associated with poor prognosis, increased invasiveness, and metastasis. Zemin Zhang has, for the first time, identified the expression of SOX4 in dysfunctional or exhausted CD4^+^ T cells across a pan-cancer T cell atlas 9. Overexpressed SOX4 enhances TGF-β signaling and downregulates ROS-driven autophagy, leading to increased CD39 expression on peripheral blood Tregs 38. Conversely, CRISPR/Cas9-mediated knockout of SOX4 produced the opposite effects. However, the role of SOX4 in regulating Treg differentiation and function within the TME remains unclear. Kuwahara *et al*. found that high expression of SOX4 protein in induced Tregs by immunoblot analysis 39. Our study reveals that SOX4 and its integrated network play a significant role in regulating the activation and inhibitory molecular characteristics of OC TI-Tregs. Flow cytometric analysis of different types of T cells in the peripheral blood and tumor tissues of OC patients confirms that SOX4^+^Tregs are the predominant type among OC TI-Tregs.

In addition, by mimicking persistent antigenic stimulation and metabolic stress experienced by Tregs in the TME through hypoxia and continuous TCR stimulation, we observed significant alterations in transcript expression of the molecule PD-1, CTLA-4, OX40, and GITR upon SOX4 knockdown via CRISPR-Cas9 in Tregs. These findings underscore the critical role of the SOX4-centered regulatory network in OC TI-Tregs. Furthermore, SOX4 knockdown also markedly reduced the transcription of FOXP3, suggesting that SOX4 may act as a pivotal transcriptional hub in OC TI-Tregs, potentially influencing their activation status and enhancing their suppressive phenotype through cooperative interactions with FOXP3 within transcriptional complexes or by modulating upstream or downstream regulatory pathways.

In the TME, the differentiation and function of Tregs are profoundly influenced by metabolic reprogramming 20. Tregs within the TME must adapt to the unique metabolic conditions imposed by the tumor, including hypoxia, nutrient deprivation, and the presence of various metabolic byproducts 40, 41. This adaptation often leads to a shift in metabolic pathways, such as enhanced oxidative phosphorylation and fatty acid oxidation, which are critical for sustaining the suppressive functions of Tregs 42. The interplay between metabolic cues and transcriptional regulation further drives the differentiation of Tregs into distinct subsets within the TME, each with specialized roles in maintaining immune tolerance and promoting tumor progression 43. OXPHOS plays a pivotal role in the differentiation and function of Tregs, particularly within TME. Tregs rely heavily on OXPHOS for energy production, especially during their differentiation into highly suppressive phenotypes 44. This metabolic pathway not only provides the necessary ATP for cellular processes but also supports the generation of ROS, which are involved in signaling pathways that reinforce the suppressive function of Tregs. The reliance on OXPHOS allows Tregs to thrive in the nutrient-deprived and hypoxic conditions of the TME, where glycolysis might be less efficient. Gene set enrichment analysis indicates that the OXPHOS pathway is significantly expressed in OC TI-Tregs 42, with SOX4's key downstream target gene MT1X being a member of the OXPHOS gene set, playing a critical role in maintaining normal mitochondrial function 45. The study reveals that MT1X 46, through its antioxidant properties, promotes mitochondrial membrane integrity and internal redox balance, thereby inhibiting mitochondrial dysfunction and ensuring a smooth electron transport chain to support the OXPHOS process. Additionally, MT1X alleviates mitochondrial damage from OXPHOS by eliminating excess accumulation of ROS, maintaining intracellular redox balance 47. Furthermore, MT1X indirectly optimizes electron transport chain function and efficiency by regulating the availability and homeostasis of copper and zinc ions. Other genes in the SOX4 target gene network, such as MT2A, IFI27, CD27, and SLC30A1, also play potential roles in Treg migration, suppression, and function. Meanwhile, qPCR further validated the high transcriptional expression of MT1X in TI-Tregs from OC. Our findings, consistent with the work of Sun *et al.* 48 demonstrating that MT-1 protein significantly expands the FOXP3⁺ population and upregulates *FOXP3* mRNA in CD4⁺ T cells, establish MT1X as a critical downstream effector of SOX4. Based on this observation, our data suggest that SOX4 coordinates a MT1X–OXPHOS regulatory axis that is essential for sustaining Treg identity. In particular, SOX4 induced MT1X expression appears to contribute to the maintenance of intracellular redox balance, as reflected by reduced intracellular and mitochondrial ROS levels in tumor-infiltrating Tregs compared with conventional CD4⁺ T cells. By simultaneously limiting oxidative stress and supporting elevated mitochondrial respiratory activity, this metabolic configuration may create a permissive condition for FOXP3 induction and stabilization. Our functional experiments also showed that SOX4 mediated FOXP3 upregulation depends on intact mitochondrial oxidative phosphorylation, as pharmacological inhibition of OXPHOS with rotenone weakened FOXP3 induction. Together, these findings suggest that SOX4 promotes a mitochondria-dependent metabolic state, partially supported by MT1X, that is essential for maintaining the Treg lineage identity.

## Conclusions

In summary, SOX4 emerges as a central regulator of functional differentiation in OC associated TI-Tregs. It promotes Treg activation and enhances the expression of inhibitory molecules while concurrently facilitating CD4⁺ T-cell polarization toward a Treg-like state through metabolic reprogramming of oxidative phosphorylation. Ultimately, SOX4 facilitates immunotolerance by promoting TI-Treg fitness within the TME, suggesting that targeting SOX4 or its upstream regulatory network may represent a potential therapeutic strategy for OC immunotherapy.

## Supplementary Material

Supplementary figures and tables.

## Data Availability

All data generated or analyzed during this study are included in this published article. The data used in the current study are available from the corresponding authors upon reasonable request.

## Abbreviations

Tregs: regulatory T cells
OC: ovarian cancer
TI-Tregs/TIL-Tregs: tumor-infiltrating Tregs
TF: transcription factor
SOX4: SRY-Box Transcription Factor 4
TME: Tumor microenvironment
SNN: Shared nearest neighbor
IFNG: Interferon-γ
HR: Hazard ratio
NK: Natural killer
DCs: Dendritic cells
OXPHOS: Oxidative phosphorylation
SOX: SRY-related HMG-box
EMT: Epithelial-mesenchymal transition
ROS: Reactive oxygen species
siRNA: Small interfering RNA
PBMCs: Peripheral blood mononuclear cells
HGSOC: High-grade serous ovarian cancer
GEO: Gene expression omnibus
TPM: Transcripts Per Kilobase of exon model per Million mapped reads
RNP: ribonucleoprotein
CREA: Creatinine
ALT: Alanine Aminotransferase
AST: Aspartate Aminotransferase
TBIL: Total Bilirubin

## References

[1] Cho K R, Shih I-M (2009). Ovarian cancer. Annu Rev Pathol.

[2] Garg V, Oza A M (2023). Treatment of Ovarian Cancer Beyond PARP Inhibition: Current and Future Options. Drugs.

[3] Wu L, Wang J, Li Q, Wang D, Zhang C, Tang J (2026). Fuzuloparib with or without apatinib as maintenance therapy in newly diagnosed, advanced ovarian cancer (FZOCUS-1): A multicenter, randomized, double-blind, placebo-controlled phase 3 trial. CA Cancer J Clin.

[4] Luo Y, Xia Y, Liu D, Li X, Li H, Liu J (2024). Neoadjuvant PARPi or chemotherapy in ovarian cancer informs targeting effector Treg cells for homologous-recombination-deficient tumors. Cell.

[5] Schoutrop E, Moyano-Galceran L, Lheureux S, Mattsson J, Lehti K, Dahlstrand H (2022). Molecular, cellular and systemic aspects of epithelial ovarian cancer and its tumor microenvironment. Semin Cancer Biol.

[6] Bagaev A, Kotlov N, Nomie K, Svekolkin V, Gafurov A, Isaeva O (2021). Conserved pan-cancer microenvironment subtypes predict response to immunotherapy. Cancer Cell.

[7] Campesato L F, Budhu S, Tchaicha J, Weng C-H, Gigoux M, Cohen I J (2020). Blockade of the AHR restricts a Treg-macrophage suppressive axis induced by L-Kynurenine. Nat Commun.

[8] You S, Li S, Zeng L, Song J, Li Z, Li W (2024). Lymphatic-localized Treg-mregDC crosstalk limits antigen trafficking and restrains anti-tumor immunity. Cancer Cell.

[9] Zheng L, Qin S, Si W, Wang A, Xing B, Gao R (2021). Pan-cancer single-cell landscape of tumor-infiltrating T cells. Science.

[10] Dura B, Choi J-Y, Zhang K, Damsky W, Thakral D, Bosenberg M (2019). scFTD-seq: freeze-thaw lysis based, portable approach toward highly distributed single-cell 3' mRNA profiling. Nucleic Acids Res.

[11] Martin M (2011). Cutadapt removes adapter sequences from high-throughput sequencing reads. EMBnet.journal 17.1.

[12] Dobin A, Davis C A, Schlesinger F, Drenkow J, Zaleski C, Jha S (2013). STAR: ultrafast universal RNA-seq aligner. Bioinformatics.

[13] Butler A, Hoffman P, Smibert P, Papalexi E, Satija R (2018). Integrating single-cell transcriptomic data across different conditions, technologies, and species. Nat Biotechnol.

[14] Satija R, Farrell J A, Gennert D, Schier A F, Regev A (2015). Spatial reconstruction of single-cell gene expression data. Nat Biotechnol.

[15] Korsunsky I, Millard N, Fan J, Slowikowski K, Zhang F, Wei K (2019). Fast, sensitive and accurate integration of single-cell data with Harmony. Nat Methods.

[16] Xu J, Fang Y, Chen K, Li S, Tang S, Ren Y (2022). Single-Cell RNA Sequencing Reveals the Tissue Architecture in Human High-Grade Serous Ovarian Cancer. Clin Cancer Res.

[17] Chu T, Wang Z, Pe'er D, Danko C G (2022). Cell type and gene expression deconvolution with BayesPrism enables Bayesian integrative analysis across bulk and single-cell RNA sequencing in oncology. Nat Cancer.

[18] You Y, Dong X, Wee Y K, Maxwell M J, Alhamdoosh M, Smyth G K (2023). Modeling group heteroscedasticity in single-cell RNA-seq pseudo-bulk data. Genome Biol.

[19] Love M I, Huber W, Anders S (2014). Moderated estimation of fold change and dispersion for RNA-seq data with DESeq2. Genome Biol.

[20] Xia L, Oyang L, Lin J, Tan S, Han Y, Wu N (2021). The cancer metabolic reprogramming and immune response. Mol Cancer.

[21] Kleshchevnikov V, Shmatko A, Dann E, Aivazidis A, King H W, Li T (2022). Cell2location maps fine-grained cell types in spatial transcriptomics. Nat Biotechnol.

[22] Shan F, Cillo A R, Cardello C, Yuan D Y, Kunning S R, Cui J (2023). Integrated BATF transcriptional network regulates suppressive intratumoral regulatory T cells. Sci Immunol.

[23] Kim S M, Shin S C, Kim E E, Kim S-H, Park K, Oh S J (2018). Simple *in Vivo* Gene Editing via Direct Self-Assembly of Cas9 Ribonucleoprotein Complexes for Cancer Treatment. ACS Nano.

[24] Miyara M, Yoshioka Y, Kitoh A, Shima T, Wing K, Niwa A (2009). Functional delineation and differentiation dynamics of human CD4+ T cells expressing the FoxP3 transcription factor. Immunity.

[25] Vasanthakumar A, Liao Y, Teh P, Pascutti M F, Oja A E, Garnham A L (2017). The TNF Receptor Superfamily-NF-κB Axis Is Critical to Maintain Effector Regulatory T Cells in Lymphoid and Non-lymphoid Tissues. Cell Rep.

[26] Levine A G, Mendoza A, Hemmers S, Moltedo B, Niec R E, Schizas M (2017). Stability and function of regulatory T cells expressing the transcription factor T-bet. Nature.

[27] Gocher-Demske A M, Cui J, Szymczak-Workman A L, Vignali K M, Latini J N, Pieklo G P (2023). IFNγ-induction of TH1-like regulatory T cells controls antiviral responses. Nat Immunol.

[28] Sjaastad L E, Owen D L, LaRue R S, Farrar M A (2020). Interferons in Treg development and function. The Journal of Immunology.

[29] Bergen V, Lange M, Peidli S, Wolf F A, Theis F J (2020). Generalizing RNA velocity to transient cell states through dynamical modeling. Nat Biotechnol.

[30] Aibar S, González-Blas C B, Moerman T, Huynh-Thu V A, Imrichova H, Hulselmans G (2017). SCENIC: single-cell regulatory network inference and clustering. Nat Methods.

[31] Toker A, Nguyen L T, Stone S C, Yang S Y C, Katz S R, Shaw P A (2018). Regulatory T Cells in Ovarian Cancer Are Characterized by a Highly Activated Phenotype Distinct from that in Melanoma. Clin Cancer Res.

[32] Chang Y, Yu J, Qiu Z, Zhang J, Yang Z, He J (2026). Regulatory T cells in ovarian cancer: Insights into cancer immunotherapy. Crit Rev Oncol Hematol.

[33] Mollaoglu G, Tepper A, Falcomatà C, Potak H T, Pia L, Amabile A (2024). Ovarian cancer-derived IL-4 promotes immunotherapy resistance. Cell.

[34] Ma C, Yang C, Peng A, Sun T, Ji X, Mi J (2023). Pan-cancer spatially resolved single-cell analysis reveals the crosstalk between cancer-associated fibroblasts and tumor microenvironment. Mol Cancer.

[35] Chowdhary K, Léon J, Mathis D, Benoist C (2024). An integrated transcription factor framework for Treg identity and diversity. Proc Natl Acad Sci U S A.

[36] Bohacova P, Terekhova M, Tsurinov P, Mullins R, Husarcikova K, Shchukina I (2024). Multidimensional profiling of human T cells reveals high CD38 expression, marking recent thymic emigrants and age-related naive T cell remodeling. Immunity.

[37] David C J, Huang Y-H, Chen M, Su J, Zou Y, Bardeesy N (2016). TGF-β Tumor Suppression through a Lethal EMT. Cell.

[38] Gerner M C, Ziegler L S, Schmidt R L J, Krenn M, Zimprich F, Uyanik-Ünal K (2020). The TGF-b/SOX4 axis and ROS-driven autophagy co-mediate CD39 expression in regulatory T-cells. FASEB J.

[39] Kuwahara M, Yamashita M, Shinoda K, Tofukuji S, Onodera A, Shinnakasu R (2012). The transcription factor Sox4 is a downstream target of signaling by the cytokine TGF-β and suppresses T(H)2 differentiation. Nat Immunol.

[40] Wang Y, Huang T, Gu J, Lu L (2023). Targeting the metabolism of tumor-infiltrating regulatory T cells. Trends Immunol.

[41] Kumagai S, Koyama S, Itahashi K, Tanegashima T, Lin Y-T, Togashi Y (2022). Lactic acid promotes PD-1 expression in regulatory T cells in highly glycolytic tumor microenvironments. Cancer Cell.

[42] Raud B, Roy D G, Divakaruni A S, Tarasenko T N, Franke R, Ma E H (2018). Etomoxir Actions on Regulatory and Memory T Cells Are Independent of Cpt1a-Mediated Fatty Acid Oxidation. Cell Metab.

[43] Delgobo M, Weiß E, Ashour D, Richter L, Popiolkowski L, Arampatzi P (2023). Myocardial Milieu Favors Local Differentiation of Regulatory T Cells. Circ Res.

[44] Du L, Lin L, Li Q, Liu K, Huang Y, Wang X (2019). IGF-2 Preprograms Maturing Macrophages to Acquire Oxidative Phosphorylation-Dependent Anti-inflammatory Properties. Cell Metab.

[45] Liu B, Hua D, Shen L, Li T, Tao Z, Fu C (2024). NPC1 is required for postnatal islet β cell differentiation by maintaining mitochondria turnover. Theranostics.

[46] Si M, Lang J (2018). The roles of metallothioneins in carcinogenesis. J Hematol Oncol.

[47] Dai H, Wang L, Li L, Huang Z, Ye L (2021). Metallothionein 1: A New Spotlight on Inflammatory Diseases. Frontiers in immunology.

[48] Sun J, Li L, Li L, Ding L, Liu X, Chen X (2018). Metallothionein-1 suppresses rheumatoid arthritis pathogenesis by shifting the Th17/Treg balance. Eur J Immunol.

