# Supplementary material for: An integrated SOX4-driven transcriptional and metabolic program governs tumor-infiltrating regulatory T cells in ovarian cancer
Source: Theranostics. 2026 Aug 12;16(15):8611–32. doi: 10.7150/thno.131793 (PMC13549143; doi:10.7150/thno.131793)
Supplement: Supplementary file 1 [file thnov16p8611s1.pdf]

Supporting Information

Lei Wu[#1,2], Ziqi Tao[#1,2], Yuelu Zhang[#1,2], Yepeng Mao[#1,2], Lei Zhang1,2,3, Lina Yan1,2,4, Rong Li1,2,4, Lingfei Zhou1,2, Zhijie Liu1,2, Wenwen Shang1,2, Shuna Liu1,2, Jianfang Lou1,2, Wenwen Shang1,2, Xi Huang1,2, Ting Wang#1,2, Fang Wang#1,2**

1Department of Laboratory Medicine, The First Affiliated Hospital of Nanjing Medical University, No. 300 of Guangzhou Road, Nanjing, 210029, China.

2Branch of National Clinical Research Center for Laboratory Medicine, Nanjing, 210029, China.

3Department of Gynecology, The Affiliated Huaian NO.1 People's Hospital of Nanjing Medical University, Huaian, 223300, China.

4Department of Gynecology, Women's Hospital of Nanjing Medical University, Nanjing, 210004, China.

[#]Contributed equally.

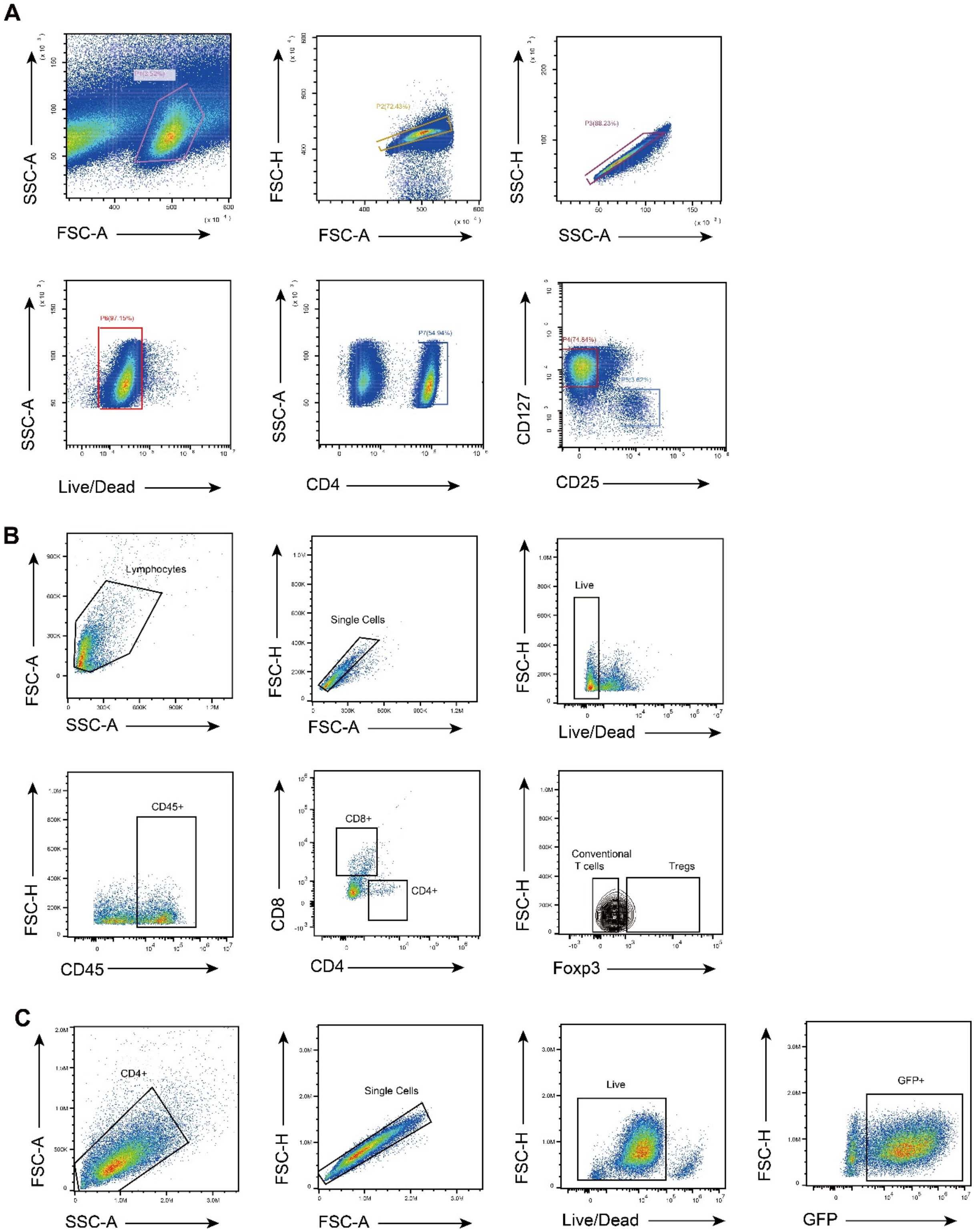

FigureS1. **Gating strategies for Treg isolation, murine immune profiling, and T-cell transduction. (A)** Gating scheme for sorting human primary $CD4^+CD25^+CD127^-$ Tregs via

sequential selection of Lymphocytes; Single Cells (FSC); Single Cells (SSC); Live Cells; $CD4^+$ T Cells; $CD25^+CD127^-$ Tregs. **(B)** Flow cytometry gating strategy for the murine ovarian cancer tumor-bearing model: Lymphocytes; Single Cells (FSC); Live cells; $CD45^+$ cells; $CD8^+$ T cells and $CD4^+$ Tcells; Conventional T cells: $CD4^+Foxp3^-$ T cells; Tregs: $CD4^+Foxp3^+$ T cells. **(C)** Transduction efficiency assessment of SOX4-overexpressing primary $CD4^+$ T cells gated on single, viable, GFP^+ $CD4^+$ T cells.

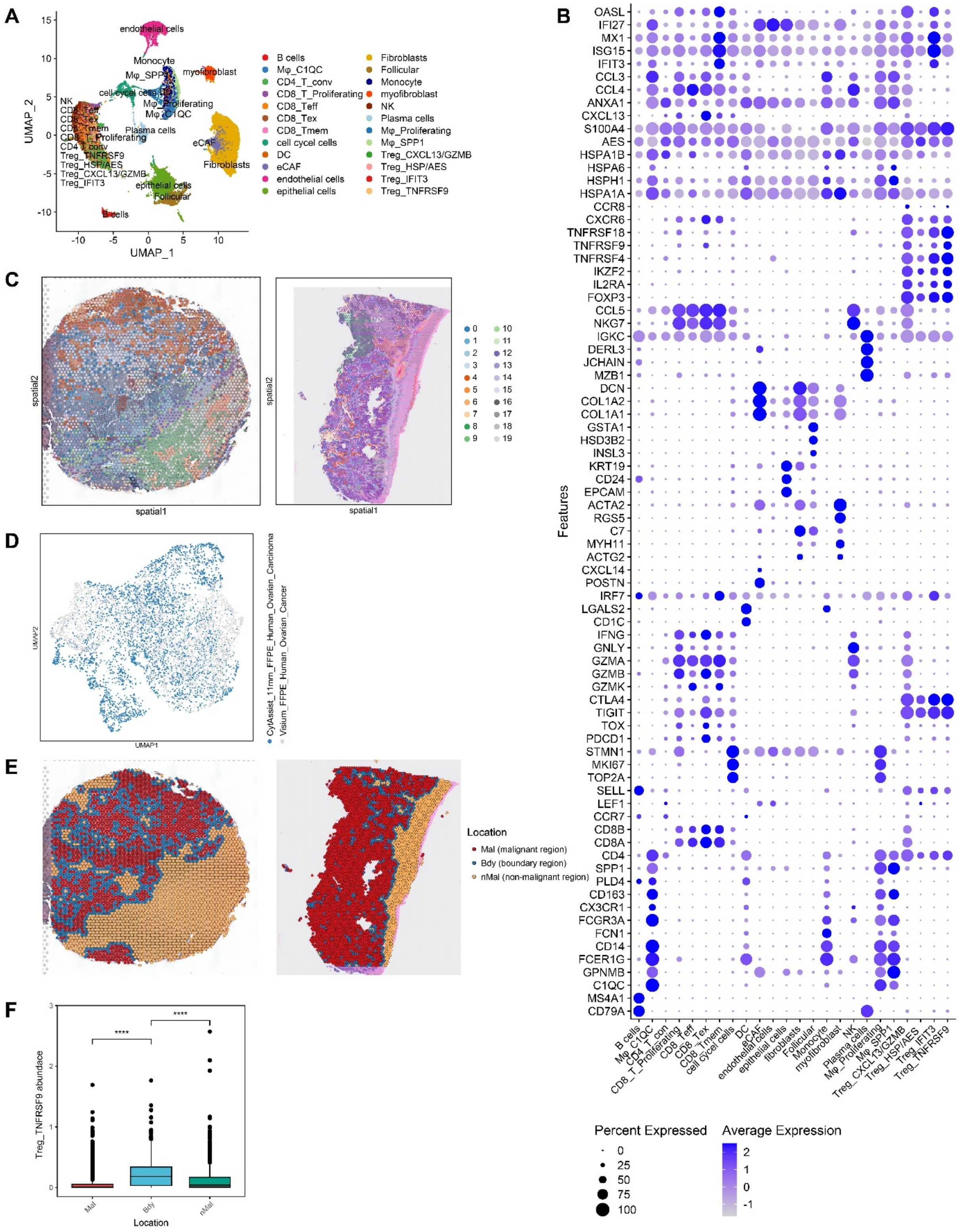

FigureS2. Spatial distribution and expression patterns of cellular subtypes in tumor and normal tissue regions. **(A)** UMAP plot showing dimensional reduction of all cells in the GSE184880 dataset. **(B)** Dot plot showing cell classification in the GSE184880 dataset. **(C-D)** Integration

of spatial transcriptomics data of ovarian cancer, with different colors representing distinct clusters. **(E)** Distribution of various cell or tissue types in the tissue section. Mal (red): malignant region, indicating diseased or tumor areas. Bdy (blue): boundary region, representing the interface between malignant and normal tissue. nMal (yellow): non-malignant region, indicating normal or unaffected tissue. **(F)** Expression abundance of the Treg_TNFRSF9 cluster across different regions in the tissue section. *****P*< 0.0001

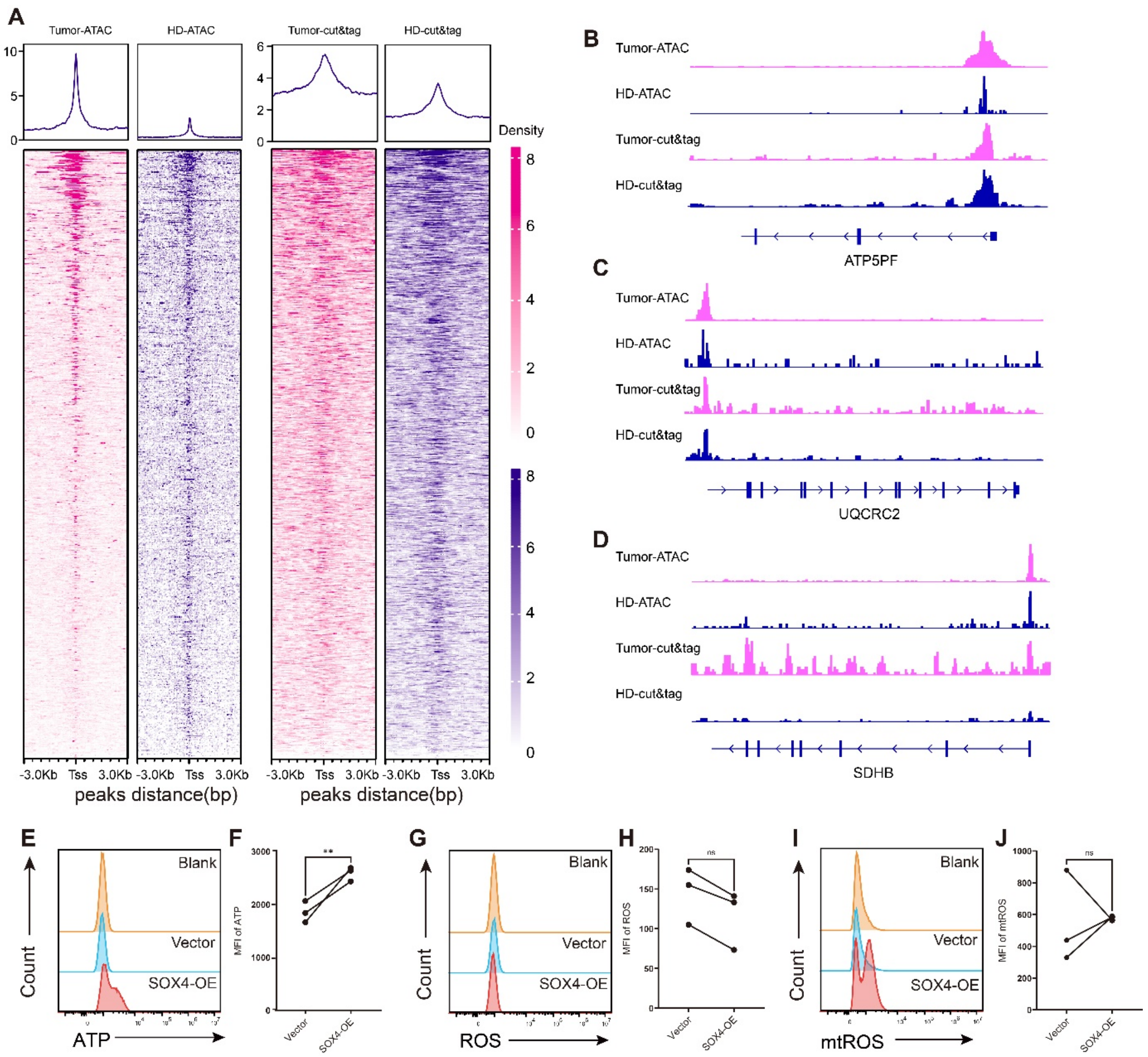

FigureS3. Chromatin accessibility and SOX4-linked metabolic readouts in Tregs. **(A)** Peak counts from ATAC-seq and CUT&Tag in ovarian tumor tissue versus healthy-donor peripheral

blood. **(B-D)** Genome-browser views of ATAC-seq and CUT&Tag signal at OXPHOS genes ATP5PF (B), UQCRC2 (C), and SDHB (D). **(E–J)** Cellular ATP, ROS, and mitochondrial ROS (mtROS) in Tregs from blank, vector, and SOX4-overexpression ($CD4^+$ T cells) groups: representative flow histograms (E, G, I) and quantification by mean fluorescence intensity (MFI) (F, H, J).

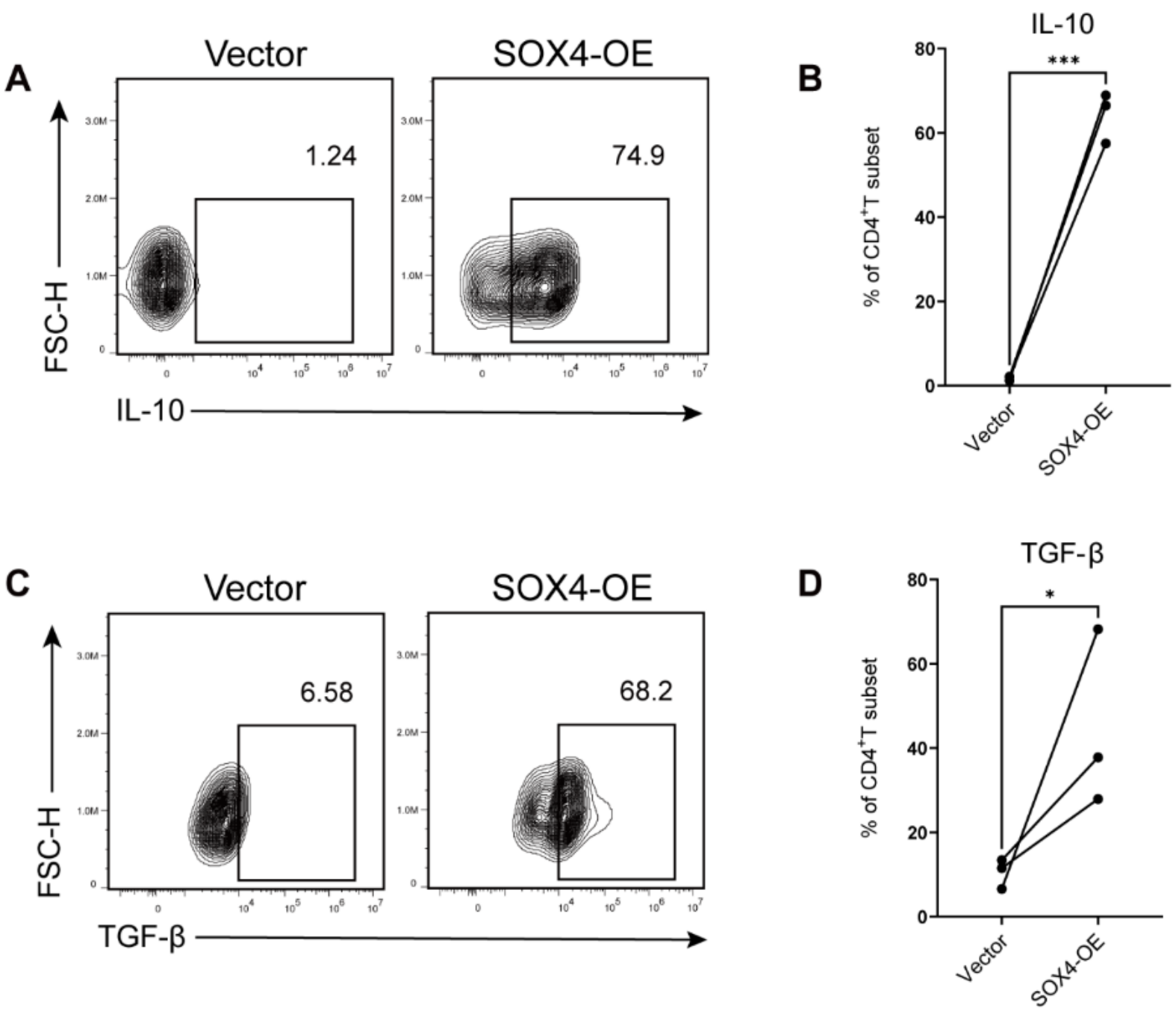

FigureS4. SOX4 induces $CD4^+$ T cells to differentiate into Tregs. **(A)** Representative flow-cytometry plots of IL-10 secretion by Tregs in control and SOX4-OE groups. **(B)** Flow-cytometry quantification of IL-10^+ Tregs in control vs SOX4-OE groups. **(C)** Representative flow-cytometry plots of TGF-β secretion by Tregs in control and SOX4-OE groups. **(D)** Flow-cytometry quantification of TGF-β^+ Tregs in control vs SOX4-OE groups.

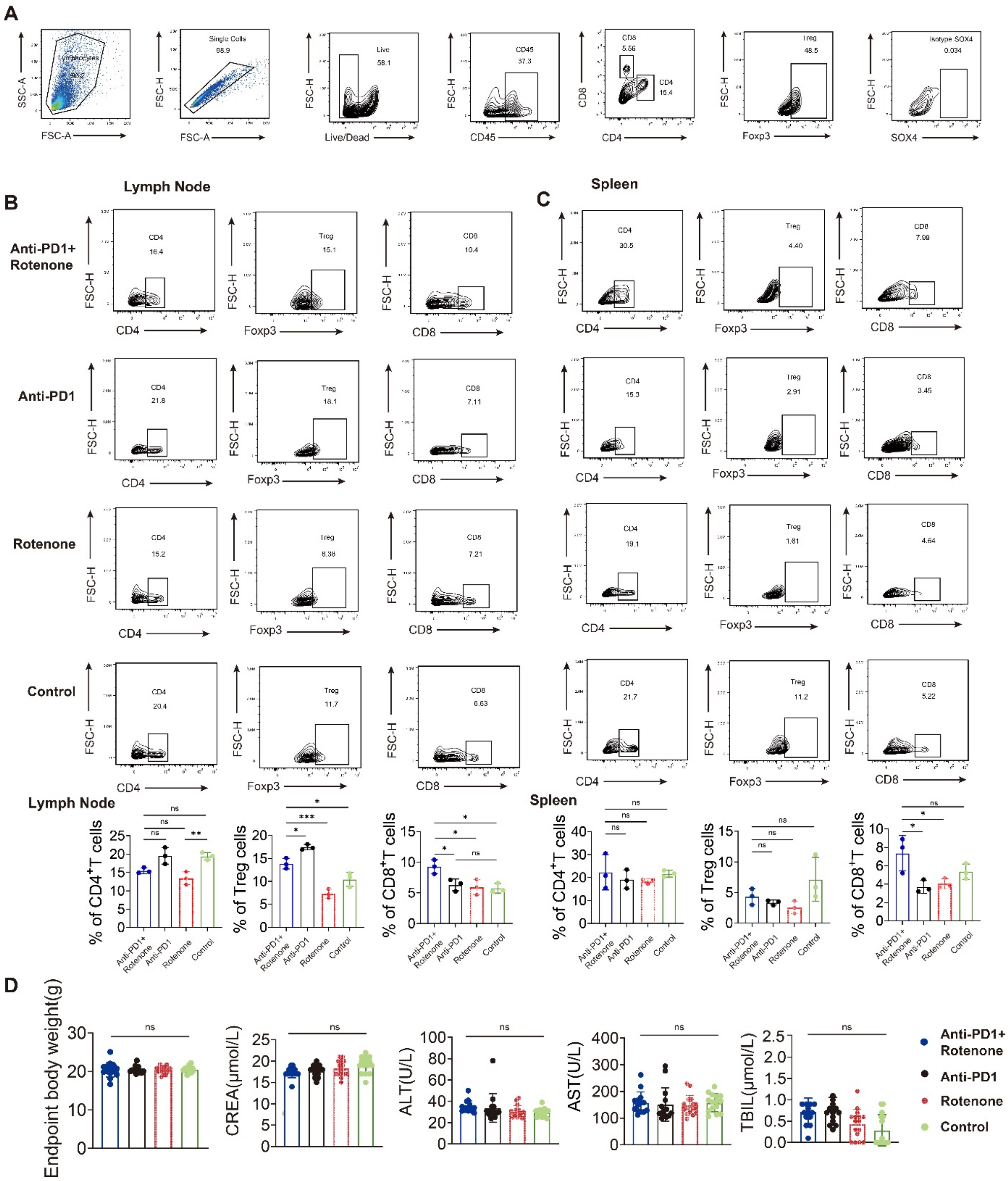

Figure S5. Gating strategy, peripheral immune profiling and systemic toxicity assessment following Anti-PD-1 and Rotenone treatment. **(A)** Representative flow cytometry gating hierarchy for identifying tumor-infiltrating lymphocytes (TILs). **(B-C)** Representative flow cytometry plots and statistical quantification of T cell subsets in the Lymph Nodes (B) and Spleen (C). $CD8^{+}$ T cells: The combination of Anti-PD-1 and Rotenone significantly increased

the percentage of $CD8^+$ T cells in both peripheral organs compared to the control and monotherapy groups. $CD4^+$ and Treg cells: No marked alterations were observed in the total $CD4^+$ T cell population across groups. However, the combination therapy and Rotenone monotherapy tended to reduce the Treg frequency in the lymph nodes. Data are presented as mean ± SD. **(D)** Physiological and biochemical parameters of mice across the four treatment groups (Anti-PD-1 + Rotenone, Anti-PD-1 monotherapy, Rotenone monotherapy, and Control). Evaluation includes endpoint body weight (g) and serum levels of Creatinine (CREA), Alanine Aminotransferase (ALT), Aspartate Aminotransferase (AST) and Total Bilirubin (TBIL). Data indicate no significant systemic toxicity or organ damage across groups (*P < 0.05; **P < 0.01; ***P < 0.001).

Table S1. Single-cell sequencing enrolled case information

Category	Age (years)	Pathology Type	Clinical Stage	Lymph Node Metastasis	Peritoneal Metastasis	Ascites	CA125 (U/mL)	CA153 (U/mL)	HE4 (pmol/L)	CA199 (U/mL)
Tumor	70	High-Grade Serous Carcinoma	IIA	negative	negative	without	131	34.3	117	17.4
	66	High-Grade Serous Carcinoma	IIIA	negative	positive	with	49.9	/	131	10.7
	60	High-Grade Serous Carcinoma	IIIC	negative	positive	with	647	83.3	374	7.88
Peritumoral tissue	56	High-Grade Serous Carcinoma	IIIC	positive	positive	with	523	23.1	921	<2
Ovarian cancer Blood	47	High-Grade Serous Carcinoma	IIA	negative	negative	without	77.06	11.36	95.38	10.16

Table S2. Clinical characteristics of paticipants

Number	Age (years)	Pathology Type	Clinical Stage	Lymph Node Metastasis	Peritoneal Metastasis	Ascites	CA125 (U/mL)	CA153 (U/mL)	HE4 (pmol/L)	CA199 (U/mL)
1	43	Endometrioid Carcinoma	IIB	/	negative	with	784.7	85.88	205.4	65.45
2	48	Clear Cell Carcinoma	IC	negative	negative	with	265.3	22.17	50.73	18.49
3	59	Endometrioid Carcinoma	IIA	negative	negative	/	14.9	15	39.2	9.2
4	68	Poorly Differentiated Neuroendocrine Carcinoma	IIIA1	positive	positive	with	57.64	4.27	86.91	4.48
5	51	Endometrioid Carcinoma	IVB	positive	positive	with	/	/	/	/
6	54	Clear Cell Carcinoma	IIA	negative	negative	without	69.87	32.87	82.5	12.49
7	51	High-Grade Serous Carcinoma	IA	negative	negative	/	/	/	/	/
8	68	High-Grade Small Cell Neuroendocrine Carcinoma	IIIC	positive	positive	with	/	/	/	/
9	47	High-Grade Serous Carcinoma	IIA	negative	negative	without	77.06	11.36	95.38	10.16
10	53	High-Grade Serous Carcinoma	/	positive	positive	/	1243	135.8	/	/
11	59	Clear Cell Carcinoma	/	positive	negative	/	142.7	7.46	47.97	14.93
12	57	High-Grade Serous Carcinoma	IIIC	positive	positive	/	1749	96.1	/	/

13	35	Low-Grade Serous Carcinoma	IIIC	positive	positive	with	/	/	/	/
14	24	Serous Borderline Tumor	/	negative	negative	with out	162	12. 6	72.7	29.3
15	43	Endometrioid Carcinoma	IIB	negative	negative	/	/	/	/	/
16	61	High-Grade Serous Carcinoma	/	positive	positive	with	108 5	140	784	
17	40	High-Grade Serous Carcinoma	/	positive	positive	with	16. 4	5.7 4	205	8.3
18	60	High-Grade Serous Carcinoma	IIB	negative	positive	with	/	/	/	/
19	61	High-Grade Serous Carcinoma	IIIC	positive	positive	/	/	/	/	/
20	70	High-Grade Serous Carcinoma	IIIC	positive	positive	with out	/	/	/	/
21	47	High-Grade Serous Carcinoma	IC	negative	negative	with	811	139	/	>100 0
22	50	High-Grade Serous Carcinoma	IIIB	negative	positive	with	/	/	/	/
23	42	Ovarian Adult-Type Granulosa Cell Tumor	IIIC	positive	positive	with	/	/	/	/
24	48	Endometrioid Carcinoma	IIIB	negative	negative	with	/	/	/	/
25	53	Clear Cell Carcinoma	IC	negative	negative	with	121	36. 8	262	136
26	48	High-Grade Serous Carcinoma	IIIC	negative	positive	with	/	/	/	/
27	65	Clear Cell Carcinoma	IC3	negative	negative	with	/	/	/	/
28	51	Clear Cell Carcinoma	IIIA1	positive	negative	/	/	/	/	/
29	47	Poorly Differentiated Squamous Cell Carcinoma	IIB	negative	positive	with	/	/	/	/
30	68	High-Grade Serous Carcinoma	IIIB	positive	positive	with	130	57. 4	66.5	5.59

31	68	High-Grade Serous Carcinoma	IIIC	negative	positive	with	2271	23.3	799	3.52
32	60	High-Grade Serous Carcinoma	IIIC	negative	positive	with	1909	72.6	218	16.3
33	52	High-Grade Serous Carcinoma	IIIC	positive	positive	with	820	83.7	388	2.43
34	54	Clear Cell Carcinoma	IC	negative	negative	with	43.8	11.4	67.3	34
35	48	High-Grade Serous Carcinoma	IIIA	positive	negative	/	50	4.86	62.5	11.6
36	51	High-Grade Serous Carcinoma	IIIC	/	positive	with	613	18.1	334	<2
37	38	Endometrioid Carcinoma	IC	negative	negative	without	162	5.56	136	89.4
38	47	Ovarian Cystic-Solid Mucinous Cystadenocarcinoma (Intestinal Type)	/	/	/	without	15.7	7.76	39.3	22.3
39	47	Clear Cell Carcinoma	/	positive	negative	/	24.8	222	58.2	19.8
40	60	High-Grade Serous Carcinoma	IIIC	positive	positive	/	48.6	14.1	171	5.19
41	52	Mature Cystic Teratoma	/	/	/	/	/	/	/	/
42	64	High-Grade Serous Carcinoma	IIIB	positive	positive	/	1210	24.7	130	16.3
43	79	Moderately Differentiated Mucinous Adenocarcinoma	/	negative	positive	with	98.5	25.6	98.6	>1000
44	47	Clear Cell Carcinoma	IIIA	positive	negative	/	24.8	9.76	58.2	19.8
45	53	Clear Cell Carcinoma	IC	negative	negative	/	121	36.8	262	136

46	48	Serous Borderline Papillary Tumor	/	negative	negative	with	104	14.7	412	16
47	52	Endometrioid Carcinoma	IC	negative	negative	/	43.6	8.42	93.6	10.9
48	53	High-Grade Serous Carcinoma	/	positive	positive	/	1243	135.8	/	/

Table S3. Antibodies used for flow cytometry

Number	Antibodies	Company	Catalog number
1	FITC anti-human CD4 Antibody	Biolegend	300506
2	Hu CD127 PE hIL-7R-M21	BD Pharmingen	557938
3	Hu CD25 APC M-A251	BD Pharmingen	555434
4	Brilliant Violet 510™ anti-human CD25 Antibody	Biolegend	302640
5	BV421 Mouse Anti-Human CD25(M-A251)	BD Pharmingen	562442
6	eBioscience™ Fixable Viability Dye eFluor™ 780	Thermo fisher	65-0865-18
7	APC anti-human CD279 (PD-1) Antibody	Biolegend	135209
8	PE/Cyanine7 anti-human CD366 (Tim-3) Antibody	Biolegend	345013
9	APC anti-human CD366 (Tim-3) Antibody	Biolegend	345012
10	APC anti-human CD152 (CTLA-4) Antibody	Biolegend	349907
11	APC anti-human CD223 (LAG-3) Antibody	Biolegend	369212
12	PE/Cyanine7 anti-human CD45 Antibody	Biolegend	368532
13	Brilliant Violet 605™ anti-human CD3 Antibody	Biolegend	317322
14	BD Horizon™ BV510 Mouse Anti-Human CD3	BD Pharmingen	564713
15	APC Mouse Anti-Human CD137(4B4-1)	BD Pharmingen	550890
16	BB700 Mouse Anti-Human GITR (CD357)(V27-580)	BD Pharmingen	747659
17	APC anti-human CD39 Antibody	Biolegend	328209
18	Brilliant Violet 421™ anti-human CD8a Antibody	Biolegend	301036
19	APC anti-human CD127 (IL-7Rα) Antibody	Biolegend	351342
20	Brilliant Violet 605™ anti-human CD134 (OX40) Antibody	Biolegend	350027
21	APC anti-human IL-10 Antibody	Biolegend	506807
22	SOX4 Antibody (PCRP-SOX4-1D6) [Alexa Fluor® 405]	Novus	NBP3-14180AF405
23	PE/Cyanine7 anti-mouse CD45 Antibody	Biolegend	157206
24	FITC anti-mouse CD4	Biolegend	100406
25	Brilliant Violet 510TM anti-mouse CD279(PD-1)	Biolegend	135241

26	Brilliant Violet 421TM anti-mouse Foxp3	Biolegend	126419
27	Treg Expansion Kit	Miltenyi Biotec	130-095-345
28	Omnipur Hyaluronidase 1 EA	Merck-Millipore	HX0514-1
29	Collagenase,TypeIV, powder	Gibco	17104019
30	Deoxyribonuclease I from bovine pancreas	Sigma-Aldrich	DN25
31	L-Glutamine	Gibco	25030081
32	CellTrace™ Violet Cell Proliferation Kit, for flow cytometry	Thermo fisher	C34571
33	Recombinant Human IL-2	Peprotech	200-02
34	TRIzol™ Reagent	Invitrogen	15596018
35	APC anti-mouse CD278(ICOS)	Biolegend	117419
36	Brilliant Violet 605TM anti-mouse CD152(CTLA-4)	Biolegend	106323
37	Sox-4(B-7)PE	Santa Cruz Biotechnology	Bsc-518016PE
38	PerCP-CyTM5.5 Rat Anti-Mouse CD8a	BD Pharmingen	551162
39	BV605 Rat Anti-Mouse CD25	BD Pharmingen	563061
40	Fixable Viability Stain 780	Thermo Fisher Scientific	565388
